# Combining wearable fNIRS and immersive virtual reality to study preschoolers’ social development: a proof-of-principle study on preschoolers’ social preference

**DOI:** 10.1093/oons/kvad012

**Published:** 2023-12-15

**Authors:** Chiara Bulgarelli, Paola Pinti, Nadine Aburumman, Emily J H Jones

**Affiliations:** Centre for Brain and Cognitive Development, Birkbeck, University of London, Malet St, London, WC1E 7HX, UK; Department of Medical Physics and Biomedical Engineering, University College London, Gower Street, London, WC1E 6BT, UK; Centre for Brain and Cognitive Development, Birkbeck, University of London, Malet St, London, WC1E 7HX, UK; Department of Medical Physics and Biomedical Engineering, University College London, Gower Street, London, WC1E 6BT, UK; Department of Computer Science, St John's Building, Brunel University London, Uxbridge, Middlesex, UB8 3PH, UK; Centre for Brain and Cognitive Development, Birkbeck, University of London, Malet St, London, WC1E 7HX, UK

**Keywords:** social preference, naturalistic neuroscience, toddlerhood, fNIRS, functional connectivity, immersive virtual-reality

## Abstract

A child’s social world is complex and rich, but has traditionally been assessed with conventional experiments where children are presented with repeated stimuli on a screen. These assessments are impoverished relative to the dynamics of social interactions in real life, and can be challenging to implement with preschoolers, who struggle to comply with strict lab rules. The current work meets the need to develop new platforms to assess preschoolers’ social development, by presenting a unique virtual-reality set-up combined with wearable functional near-infrared spectroscopy (fNIRS). As a proof-of-principle, we validated this platform by measuring brain activity during self-guided social interaction in 3-to-5-year-olds, which is under-investigated, yet crucial to understand the basis of social interactions in preschoolers. 37 preschoolers chose an interaction partner from one of 4 human-like avatars of different gender and age. We recorded spontaneous brain fluctuations from the frontal and temporoparietal regions (notably engaged in social-categorization and preference) while children played a bubble-popping game with a preferred and an assigned avatar. 60% of the participants chose to play with the same-gender and same-age avatar. However, this result was driven by females (>80% vs. 50% in males). Different fronto-temporoparietal connectivity patterns when playing with the two avatars were observed, especially in females. We showed the feasibility of using a novel set-up to naturalistically assess social preference in preschoolers, which was assessed at the behavioural and functional connectivity level. This work provides a first proof-of-principle for using cutting-edge technologies and naturalistic experiments to study social development, opening new avenues of research.

## INTRODUCTION

A child’s social world is dynamic, complex and rich, resulting in multiple core neuroscientific questions about social cognitive development. Developmental neuroscientists have traditionally assessed social brain functions with conventional scientific experiments where children are presented with repeated stimuli on a screen. However, these traditional assessments are often far from the complexity and the dynamics of social interactions in real life, and passive stimuli presentation does not promote participants’ active engagement with the task [1, 2], with the added risk of evoking impoverished behavioural and neural responses. While the use of traditional experiment set-ups has been proven to be efficient with developmental populations who are able to comply with standard lab settings, typically very young infants or school-age children, this has limited our understanding of development in the preschool period. As such, very little is known about preschoolers’ social development, who struggle to comply with rigid lab rules of sitting still and paying attention to repeated stimuli on a screen for a long time.

Recent advances in technology and analytics have made it possible to test children during more naturalistic conditions, meeting the need for more dynamic and ecologically valid studies. This has enabled us to rethink the way in which we assess social development [3–6]. New methods are being developed to realistically investigate social skills in preschoolers and children, and therefore evoke more naturalistic responses from participants [7]. Although some have focused on live interactions between social partners [7–9], this approach allows very little experimental control and makes it very challenging to tease apart complex causal pathways between the behaviour of interacting partners. An alternative hybrid approach is to use a virtual-reality set-up, where experimenters can design child-friendly scenarios involving human-like avatars interacting with participants, providing a realistic yet controlled set-up to assess preschoolers development. Moreover, wearable neuroimaging tools can be easily implemented in the virtual-reality environment, allowing for the recording of neural activity in the immersive space, as well as overcoming the abovementioned challenges of gathering neuroimaging data in children in restrained contexts, such as in a fMRI scanner or tethered to stationary machines [10]. To the best of our knowledge, there are no investigations of social skills development in toddlerhood using virtual-reality set-up.

In this study, we aim to present a new platform combining wearable functional near-infrared spectroscopy (fNIRS) with the Cave Automatic Virtual Environment (CAVE) set-up for the study of social development in preschoolers. The CAVE is a unique virtual-reality room able to simulate real-world surroundings. Virtual scenarios are projected on the three walls surrounding the participant and the floor. The CAVE set-up is particularly suited and engaging for children, avoiding those issues related to typical VR headsets (e.g. not child-sized, motion sickness, heavy, etc.). fNIRS is a child-friendly neuroimaging tool that uses light in the near-infrared range to measure the changes in haemoglobin concentration in the cerebral blood flow as a proxy for brain activation [11]. The use of fNIRS with developmental populations has significantly grown over the past decades, as infants and preschoolers have thinner scalp and skull than adults, allowing the near-infrared light to effectively reach the outer layer of the cortex [12]. Moreover, recent advances in technologies made this tool fully wearable, opening up new avenues of research where participants can move around while wearing the fNIRS cap [13–16].

As a proof-of-principle, we have used this platform to investigate the development of social preferences and their neural correlates in preschoolers. To date, there are no empirical studies that investigate partner preference in toddlerhood, although this could provide information about the emergence of social understanding in early childhood. Moreover, this could have a methodological relevance, as future studies testing preschoolers could design more appropriate stimuli for use with young children. Understanding the brain processes that underlie social choice is also important, because the amount of self-initiated interactions and behaviours considerably grow during toddlerhood, yet the mechanisms underpinning their development have yet to be fully understood.

There is an extensive literature investigating infants’ preferences in social partner, suggesting that newborns look longer at attractive compared to unattractive faces [17], and female compared to male faces, regardless of the infant’s sex [18] (although this might be influenced by the gender of their primary caregiver). Infants younger than 1 year of life prefer to interact with individuals they perceive more similar to them [19–21], especially if this social partner speaks the same language [22]. Notably, most of these investigations assessed infants’ social preferences by using stimuli with female faces or voices and modulating other in-group/out-group variables of interest [19].

Understanding how partner preference during social interactions changes with age is fundamental to inform our understanding of children’s social development. As children grow, they gain more control over their interactions, making it important to identify the factors that influence their social choices. While infants primarily interact with adults, children between 3 and 5 years old often spend their days interacting with peers, especially if they attend day-care. During this period, playing in groups and cooperation between peers become more prevalent [23]. Children also start to develop knowledge of their own social characteristics, such as their gender group [24], and begin using gender-specific terms in their speech [25]. Interestingly, toddlers begin to segregate into gender-based groups during their social activities [26]. This coincides with a critical period in a child's social life, around the age of 2, when they show evidence of self-recognition sometimes interpreted to mean that children become able to differentiate between themselves and others [27, 28]. This ability co-occurs with the development of several social skills and the capability to categorize others based on similarities and differences [29]. Based on the principles of familiarity and self-similarity governing social preferences, it is expected that preschoolers will prefer to interact with peers of the same gender, in contrast to infants' preference for interacting with female adults. Additionally, gender differences in behaviour may also emerge during this period. Girls tend to exhibit more empathy and prosocial behaviour from early childhood [30–32] (although there are extensive debates on the degree to which this is biologically vs. environmentally driven [33–35]), potentially making girls more inclined to interact with other girls who have similar advanced social skills. Girls may also show greater sensitivity to the gender or age of a social partner earlier than boys due to their heightened sensitivity to social information. These gender differences in social skills appear to increase with age and are supported by different neural processes in adulthood [36, 37].

When considering the neural correlates of partner choice, adult studies highlight the role of the medial prefrontal cortex (MPFC) and temporoparietal junction (TPJ). The MPFC shows differential activity during social categorization, with greater activation for in-group versus out-group stimuli [38, 39], and when adults are asked to express social preferences [40]. Both the MPFC and the TPJ are activated when interacting with in-group members, as a possible marker of self-comparison with the in-group features, not only in adults [41], but also in infants [20]. Interestingly, the MPFC and the TPJ regions belong to the default mode network (DMN), which has been associated with psychological self-related processes, such as self-reflection and self-comparison [42, 43]. While this is well-established in adults, we have recently found that MPFC-TPJ connectivity in the low-frequency range is stronger already in 18-month-olds who have more advanced self-processing (as indexed by successful self-recognition), suggesting that the DMN might support self-related processes already after the second year of life [28]. Thus, it is expected that MPFC-TPJ functional connectivity (FC) would also be heightened during the interaction with a preferred partner in toddlers.

The overall goal of this work is to validate a unique and novel CAVE/fNIRS set-up for the study of preschoolers’ social development, by assessing for the first time preschoolers’ social preference. We tested forty-one 3-to-5-year-olds in the world’s first ToddlerLab CAVE facility, while wearing a wearable fNIRS system to measure their spontaneous brain activity. Participants were exposed to 4 human-like avatars of different gender (male, female) and age (adult, child), and asked to choose a virtual partner to play a bubble-popping game with. During the task in the CAVE, children wore a wearable fNIRS cap recording spontaneous fluctuations in oxygenated (HbO_2_) and deoxygenated blood (HHb) from the frontal and the temporo-parietal cortices while interacting with a preferred (*preferred avatar condition*) and a randomly assigned human-like avatar (*assigned avatar condition*). We hypothesize that: i) preschoolers will choose to interact with human-like avatars of same age and gender as themselves; ii) preschoolers will pop more bubbles in the *preferred* compared to the *assigned avatar* condition, indexing social preferences; iii) MPFC-TPJ connectivity in the low-frequency range will be stronger in the *preferred* compared to the *assigned avatar* condition, indexing social preferences^1^. As it is plausible to hypothesize that partner preference might change between males and females and younger and older preschoolers, we will also explore any effect of gender and age on the choice of the avatar.

This work provides a proof-of-principle for using wearable neuroimaging and immersive virtual-reality set-up for the study of social preference in preschoolers. It is therefore extremely novel as we used cutting-edge technologies to investigate preschoolers social development and test alternative methods from the standard assessments employed so far, opening up new avenues for future studies in the field of developmental neuroscience. Additionally, findings from this study will have not only theoretical implications, by informing on changes on social partner preference from infancy to childhood, but also a methodological one, by providing contextual information that should be used to guide choices of interactive partners when assessing preschoolers’ social development.

**Figure 1:**
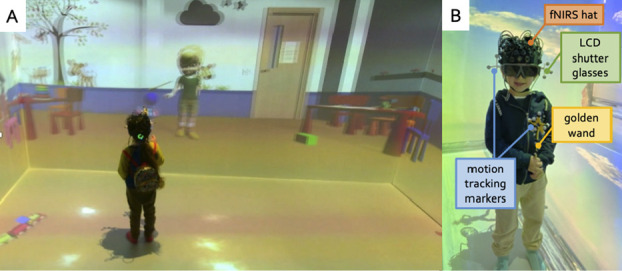
(**A**) A participant playing a bubble-popping game in the CAVE lab. (**B**) Equipment worn by a participant.

## MATERIALS AND METHODS

### Participants

Thirty-seven 3-to-5-year-olds were enrolled in the study (22 males, age mean ± SD = 4.42 ± 0.79 years) and chose a social partner among the virtual characters. 4 additional participants were recruited but refused to wear the fNIRS hat and do the task (1 male, age mean 4.07 ± 0.34 years). Among these 37 participants, 5 additional participants were excluded because: (i) the fNIRS cap did not fit on their head (3 participants, 3 males, age mean 4.77 ± 0.93 years); (ii) experimental error (1 participant); (iii) excessive motion and noise in the data (1 participant). As a result, thirty-two 3-to-5-year-olds were included in the fNIRS analyses (20 males, age mean ± SD = 4.41 ± 0.83 years). All included participants were born full-term, healthy and with normal birth weight. Participants were excluded from recruitment if they had a known significant neurodevelopmental condition or a medical condition that was likely to impact brain development or impede the child's ability to participate in this study. Written informed consent was obtained from the toddler’s caregiver prior to the start of the experiment. Ethical approval for this study was given by the Ethics Committee of the Department of Psychological Sciences, Birkbeck, University of London (No. 2122056).

### The cave automatic virtual environment (CAVE)

The experiment took place in the immersive Cave Automatic Virtual Environment at the Birkbeck ToddlerLab.

The CAVE system used in this study is a four-sided custom-designed display system (Mechdyne Corporation) with three projection walls (front and sides) and a projection floor. The front display surface measures 4.3 × 2 m; the side displays are 2.4 × 2 m; the floor is 4.3 × 2 m. Two overlapped (65%) and blended single chip laser projectors are used on the front wall and floor display surfaces, each with a resolution of 2716 × 1528 (total resolution = 3297 × 1528 pixels). On the side walls a single laser projector is used (resolution 2716 × 1528) (Fig. 1A).

Participants wore custom-built child-sized LCD (i.e. liquid crystal display) shutter glasses that enabled active-stereo viewing to increase the immersiveness of the experience in the CAVE. The CAVE is equipped with four six-degree-of-freedom optical motion-tracking cameras (Vero 1.3 X, Vicon) placed at the top corners of the CAVE. Passive markers for head tracking were attached were attached to the glasses to reorient and rotate the virtual scenes according to the participant’s head position. Motion tracking markers were also attached onto a plastic magic wand to allow participants to interact with virtual objects in the scene (i.e. to pop virtual bubbles in this case) (Fig. 1B).

### Virtual avatars

**Figure 2:**
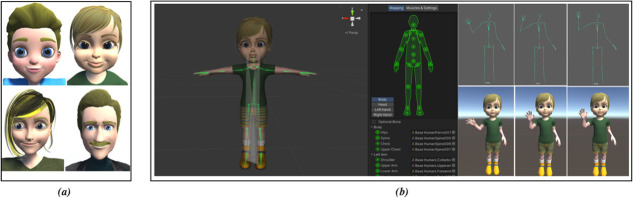
The 4 human-like avatars used in this study. (**A**) The 4 human-like avatars used in this study with a smiling facial expression. (**B**) The animation pipeline for the toddler-girl avatar using humanoid rigged and motion capture data of a waving “hello” motion

In this experiment, we employed avatars with a cartoon appearance rather than using photo-realistic avatars, as cartoon-like avatars tend to increase co-presence and avoids the *uncanny valley* effect [44] (Fig. 2). Moreover, it has been shown that children prefer more familiar and simplistic rendering styles than realistic-looking virtual humans [45]. We acquired the avatars from CGTrader (https://www.cgtrader.com), and we used Autodesk Maya (https://www.autodesk.com) to edit and customize them. For animating the facial expressions, blend shape deformation was utilized, while the motion capture database from Carnegie Mellon University has been used to animate the avatars’ bodies. To model the nonverbal communication (such as eye contact, eye blinking, and head direction), we programmed the virtual avatar’s head to follow the participant’s location or the direction of the participant’s gaze. This was done using the avatar's skeletal rig and based on the motion-tracked location, which was attached to the participant’s head. Moreover, we used a 9-frame blink duration model (at 30 frames per second) to simulate spontaneous eye blinking.

**Figure 3:**
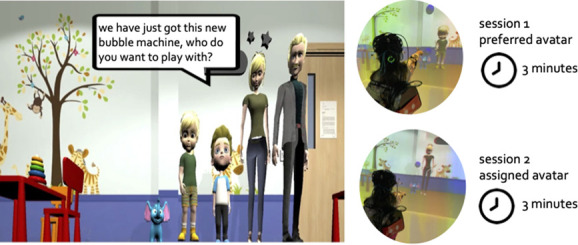
The 4 avatars in the virtual classroom with the bubble machine (the blue elephant) and the playing sessions with the preferred and assigned avatar.

### Stimuli and procedure

At the beginning of the experiment, the participant was made familiar with the CAVE set-up and the equipment. We allowed the parent and the toddler to explore the fNIRS cap and the CAVE room for as long as they wished. The parent informed the experimenter when their child was ready to wear the equipment and begin the testing session. While there was variability in the time participants took to acclimate to the equipment—with some individuals requiring more time than others—in general, participants were ready to commence the study after approximately 5 minutes of exploring the equipment. After the cap and the shuttered glasses were fitted on the participant’s head, a golden wand to pop virtual bubbles was given to the participant (The same golden wand was present in the virtual scenario to represent the participant’s wand, and it moved in sync with the participant's real wand).

The VR task was implemented using Unity (v 2019.4.18f1) in conjunction with a CAVE-specific plugin (getReal3D, Mechdyne) to allow every image to be synchronized across all the projectors. The task was set in a virtual classroom as children of this age are familiar with such environment from their daily life experience (Fig. 3).

Initially, the participant had some time to explore the virtual classroom and pop some bubbles with the wand. The time spent on this phase varied among participants, averaging approximately 3 to 5 minutes. This duration depended on the participants' preference to explore the virtual classroom and the time needed for them to successfully pop the bubbles with the magic wand. Once the participant was confident in navigating the virtual scenario and using the wand, s/he was presented with 4 human-like avatars (a woman, a man, a boy, and a girl) in the classroom. The 4 avatars introduced themselves and asked what was the child’s name. Then one avatar (randomly chosen each time) asked the participant “we have just got this new bubble machine, who do you want to play with?”, and the experimenter recorded the participant’s choice. The participant played for 3 minutes with the chosen avatar (*preferred avatar condition*). Thereafter, the participant was asked to play for 3 minutes with an avatar randomly assigned from those not chosen (either opposite gender or different age) (*assigned avatar condition*) (Fig. 3). As all participants underwent these two conditions in the same order, this might create order effect. However, asking participants to choose an avatar to play with but then making them play with another one would have been less naturalistic and could possibly be upsetting or confusing for the children. Participants who agreed to keep the equipment on after the first two conditions (N = 21), were asked to play again with the preferred avatar for 3 minutes (*preferred avatar condition 2*). Additionally, comparing the FC patterns in *preferred avatar condition 2* with the *assigned avatar condition* allowed us to more confidently rule out any order effect on the fNIRS results. Number of bubbles popped by the participant and by the avatar in each condition was recorded. As a previous study from our team with participants of similar age showed that FC reaches stability after 2 minutes [46], we collected data for 3 minutes for each condition will provide a good estimation of FC.

To make the task more realistic and interesting for the participants, the avatars played with the game and with the participants, regardless the condition (*preferred* or *assigned avatar*). In all the conditions, the avatars could pop a bubble themselves every 6-to-8 bubbles (if this was not popped by the participant). Moreover, a jingling star appeared in the virtual scenario every time the participant popped a bubble, and all the avatars rewarded the participant’s performance every 20/30 seconds with sentences like: “well done, keep going!”, “popping bubbles is such good fun!”, “you are nearly there, the bubbles are almost finished!”.

### fNIRS data acquisition

fNIRS data were recorded using two mobile systems (dual Brite MKII, Artinis Medical Systems BV, Netherlands), which use two continuous wavelengths of near-infrared light (763 nm and 841 nm) to measure changes in HbO_2_ and HHb concentration, at a sampling rate of 25 Hz. Each device was equipped with 10 light sources and 8 detectors.

**Figure 4:**
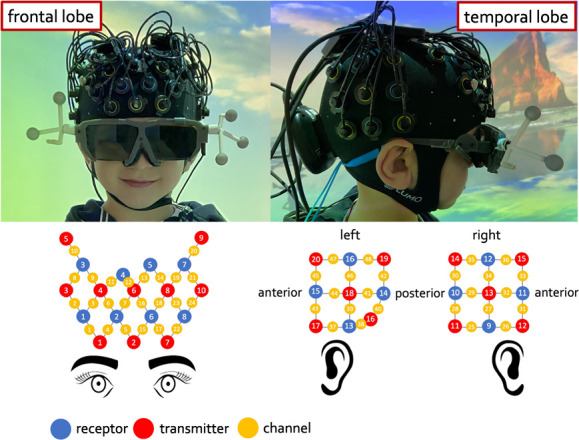
A child wearing the fNIRS hat and the fNIRS array configuration

The NIRS optodes were fitted in a flexible cap (EasyCap) made of soft neoprene. One NIRS system covered the frontal lobe, while the other system covered the temporo-parietal regions bilaterally, for a total of 48 channels (24 over the dorsal and medial frontal regions and 12 over each temporo-parietal regions). Source-detector (S-D) separation was about 25 mm, except for channel 12 over the front and channel 37 over the left temporal lobe that had a SD separation of 10 mm. We refer to these two channels as short-separation channels (SSC), and their signal was used to regress out systemic changes from the signal of the other channels (see fNIRS data processing section) (Fig. 4).

Both fNIRS systems are equipped with accelerometers within the main unit of the device. Therefore, one was attached on the back of the cap to record the participant’s head movement, while the second system was stored in a small backpack wore by the participant to avoid putting an excessive weight on the child’s head.

### fNIRS data processing

Data analyses were carried out using in-house codes developed in MATLAB (MathWorks, Natick, MA). Raw intensity data were converted to optical density (*hmrIntensity2OD.m* function from Homer2 tool [47]). Hereafter motion artefacts were corrected using wavelet (iqr = 0.8, *hmrMotionCorrectWavelet* function from Homer2). Low-quality channels, based on physiological indicators of quality were pruned using QT-NIRS (https://github.com/lpollonini/qt-nirs). The software takes advantage of the fact that the cardiac pulsation is recorded in addition to the haemodynamic activity [48], and quantifies its strength in the spectral and temporal domains with two measures: the Scalp Coupling Index (SCI) and the Peak Spectral Power (PSP) [49]. For each participant, data quality was assessed channel-by-channel and SCI and PSP were calculated every 3-second non-overlapping window (threshold SCI = 0.60, threshold PSP = 0.06, empirically defined) [49]. Channels that had both SCI and PSP below threshold for more than 60% of the windows [49], were excluded from further pre-processing steps. Hereafter the surviving channels underwent visual inspection, and channels with clear signs of noise or saturation were additionally removed. Optical density data were then bandpass filtered (0.009–0.08 Hz) (*hmrBandpassFilt* function from Homer2) and converted to relative concentrations of haemoglobin using the modified Beer–Lambert law (DPF = 5.4, 4.6) [50, 51] (*hmrOD2Conc* function from Homer2). Last, to account for the systemic changes and the effect of motion that is well known to contaminate fNIRS signal [52], especially in freely-moving participants [53], we regressed out the signal of both the SSC and the accelerometer data from the signal of all the other 46 channels. Hereafter, channels that survived pre-processing were averaged into 10 regions of interest (ROIs) (left and right medial prefrontal cortex, MPFC; left and right dorsolateral prefrontal cortex, DLPFC; left and right temporo-parietal junction, TPJ; left and right middle and superior temporal gyrus, M/STG; left and right inferior parietal lobule, IPL) following the co-registration of each participant’s array onto an age appropriate MRI template (see Co-registration of the fNIRS array section). The correlation matrix between the ROIs was calculated for both HbO_2_ and HHb for each participant, resulting in a 10 × 10 matrix of ROIs-pair correlations (R-values). We then applied Fisher z-transformation on the correlation matrix for further statistical analyses.

**Table 1:** Co-registration of each channel of the fNIRS array

Frontal Lobe			Temporo-parietal Lobe		
Channel No.	LPBA label	ROI	Channel No.	LPBA label	ROI
1	Right inferior frontal gyrus	/	25	Right middle temporal gyrus	Right M/STG
2	Right middle frontal gyrus	Right DLPFC	26	Right middle temporal gyrus	Right M/STG
3	Right middle frontal gyrus	Right DLPFC	27	Right middle temporal gyrus	Right M/STG
4	Right middle frontal gyrus	Right MPFC	28	Right angular gyrus	Right TPJ
5	Right superior frontal gyrus	Right MPFC	29	Right angular gyrus	Right TPJ
6	Right middle frontal gyrus	Right MPFC	30	Right middle temporal gyrus	Right M/STG
7	Right superior frontal gyrus	Right MPFC	31	Right superior temporal gyrus	Right M/STG
8	Right middle frontal gyrus	Right DLPFC	32	Right supramarginal gyrus	Right IPL
9	Right middle frontal gyrus	Right DLPFC	33	Right superior temporal gyrus	Right TPJ
10	Right middle frontal gyrus	Right DLPFC	34	Right angular gyrus	Right TPJ
11	Right superior frontal gyrus	Right MPFC	35	Right angular gyrus	Right IPL
12 (SSC)	Right superior frontal gyrus	/	36	Right angular gyrus	Right IPL
13	Left superior frontal gyrus	Left MPFC	37 (SSC)	Left middle temporal gyrus	/
14	Left middle frontal gyrus	Left MPFC	38	Left middle temporal gyrus	Left M/STG
15	Left superior frontal gyrus	Left MPFC	39	Left middle temporal gyrus	Left M/STG
16	Left middle frontal gyrus	Left MPFC	40	Left angular gyrus	Left TPJ
17	Left middle frontal gyrus	Left MPFC	41	Left angular gyrus	Left TPJ
18	Left middle frontal gyrus	Left MPFC	42	Left angular gyrus	Left M/STG
19	Left middle frontal gyrus	Left DLPFC	43	Left supramarginal gyrus	Left TPJ
20	Left middle frontal gyrus	Left DLPFC	44	Left supramarginal gyrus	Left IPL
21	Left middle frontal gyrus	Left DLPFC	45	Left superior temporal gyrus	Left M/STG
22	Left inferior frontal gyrus	/	46	Left angular gyrus	Left TPJ
23	Left middle frontal gyrus	Left DLPFC	47	Left angular gyrus	Left IPL
24	Left middle frontal gyrus	Left DLPFC	48	Left supramarginal gyrus	Left IPL

The table shows anatomical labels (LPBA40 atlas) and ROI associated with each channel.

First, we performed one-sample t-tests on both HbO_2_ and HHb to assess FC in the *preferred* and *assigned avatar* condition. Then we tested whether FC is greater in the *preferred avatar* condition than the *assigned avatar* condition using a paired t-test on the HbO_2_ and HHb signals separately. To ensure statistical reliability, significant results of FC between frontal and temporo-parietal channels from one sample and paired t-tests were corrected for multiple comparisons using the False Discovery Rate (FDR) method [54, 55].

### Co-registration of the fNIRS array

The 48 channels were co-registered onto a 5-year-old MRI template using a combination of the toolbox STORM-Net, functions from DOT-HUB and in-house codes.

**Figure 5:**
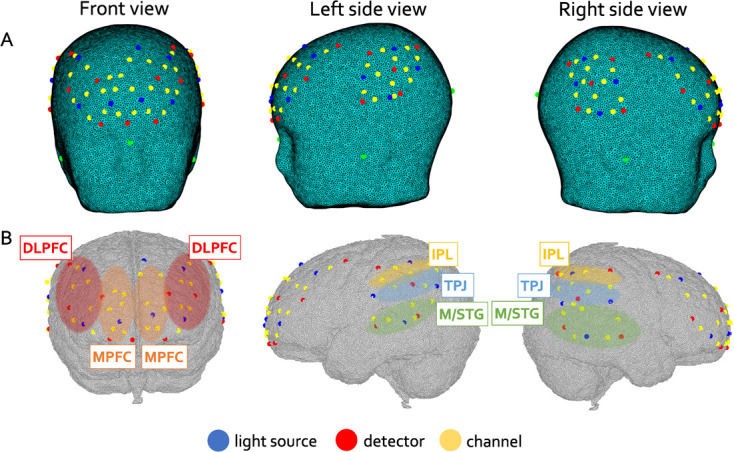
(**A**) Representation of the channels on a 5-year-old multilayer head model mesh. (**B**) Schematic representation of the channels overlapped onto the grey matter mesh extracted from the multilayer volumetric template model. ROIs are highlighted: orange represents mPFC, red represents DLPFC, green represents M/STG, blue represents TPJ, yellow represents IPL

First, reference points, optodes and channels coordinates were estimated for each participant using STORM-Net (https://github.com/yoterel/STORM-Net). This tool estimates the position of the fNIRS optodes on the participant’s scalp, by locating some reference points in a video of the participant’s wearing the cap against an ideal cap placement onto a head model. Here, we have 3D printed a 5-year-old head model of the MRI template used for the optode coregistration [56] for the offline stage of STORM-NET. Then, a 5-year-old MRI template from the Neurodevelopmental MRI Database of the University of South Carolina (http://jerlab.psych.sc.edu/NeurodevelopmentalMRIDatabase/) was segmented into 5 tissue types (scalp, skull, cerebrospinal fluid, grey matter (GM), white matter) and used to generate a volumetric multilayer mask. Routines from the DOT-HUB toolbox (https://github.com/DOT-HUB) employing iso2mesh [57] were used to create a tetrahedral volumetric mesh, as well as a mesh of the brain (i.e. GM tissue type). For each participant, the MRI mesh model was registered on the participant’s head through an affine transformation using the model’s cranial landmarks (Nasion, Inion, Right and Left Preauricular points, Cz) and the same subject-specific landmarks estimated by STORM-NET. The affine transformation was applied to the GM mesh. The channels coordinates derived from STORM-NET were first co-registered onto the scalp mesh and then projected onto the GM surface mesh. A 1.25 cm radius sphere centred around each channel’s coordinate on the GM mesh was created to evaluate the anatomical regions overlapped by each channel. To this goal the LPBA40 atlas was used, and anatomical labels were assigned to each channel considering a minimum percentage of overlap to that region of 25%. Channels were then assigned to one of the 10 ROIs based on the anatomical label and the spatial location on the brain (see Table 1). Figure 5 provides a graphical representation of the optodes location on the template head and the brain areas covered by the fNIRS array used, where the ROIs are colour coded.

**Figure 6:**
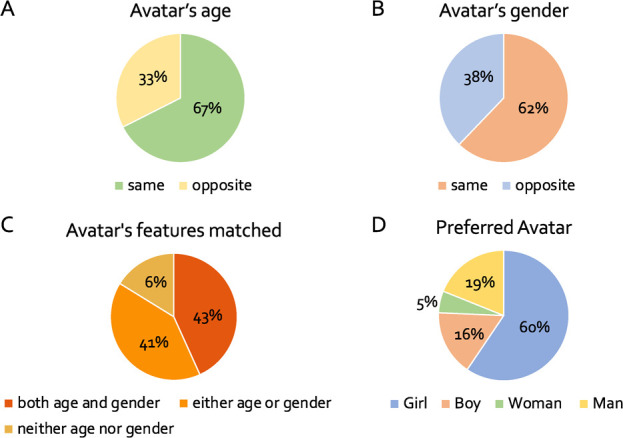
(**A**) Number of participants that chose an adult (opposite) or toddler avatar (same). (**B**) Number of participants that chose an avatar of the same or opposite gender. (**C**) Avatar’s features (age and gender) matched. (**D**) No of choices as preferred avatar

## RESULTS

### Behavioural results

37 preschoolers in total contributed to the behavioural data. 25 out of 37 participants (67%) chose to play with an avatar of the same age (Fig. 6A), and 23 out of 37 participants (62%) chose to play with an avatar of the same gender (Fig. 6B). 16 participants (43%) chose an avatar that matched both their gender and age, while 15 participants (41%) chose an avatar that matched only their gender or age, and 6 (16%) neither their gender nor their age (Fig. 6C). Out of the 37 preschoolers, 22 (60%) preferred to play with the girl-avatar, 6 (16%) with the boy-avatar, 2 (5%) with the woman-avatar and 7 (19%) with the man-avatar (Fig. 6D).

**Figure 7:**
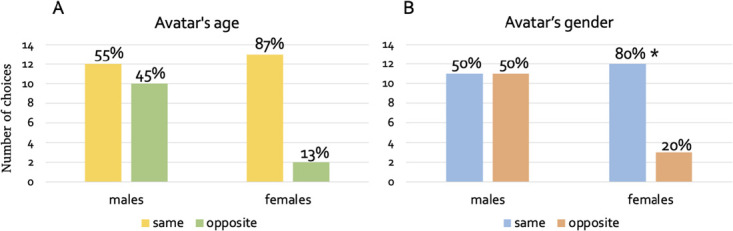
(**A**) Bar plots showing toddler (same age) and adult (opposite age) avatar chosen by males and females. (**B**) Bar plots showing same and opposite-sex avatar chosen by males and females. ^*^ indicates statistically significant difference at the chi-square (p<0.05)

We also explored whether there were any participants’ gender- or age-related differences in choosing the avatar. Out of the 22 male participants, 12 (55%) chose to play with a toddler-avatar and 10 (45%) chose to play with an adult avatar. Out of the 15 female participants, 13 (87%) chose to play with a toddler-avatar and only 2 (13%) chose to play with an adult avatar. A chi-square test of independence showed that there was a statistically significant association between the participant’s gender and the preferred avatar’s gender (*X^2^*(1, *N* = 37) = 4.1, *p* = 0.04) (Fig. 7A). Out of the 22 male participants, 11 (50%) chose to play with a same- gender avatar and 11 (50%) chose to play with an opposite-gender avatar. Out of the 15 females participants, 12 (80%) chose to play with a same- gender avatar and only 3 (20%) chose to play with an opposite-gender avatar. A chi-square test of independence showed that there was a trend towards a significant association between the participant’s gender and the preferred avatar’s age (*X^2^*(1, *N* = 37) = 3.5, *p* = 0.058) (Fig. 7B). To sum up, females only preferred to play with the same-gender and same-age avatar, although just the association between participants’ gender and choice of avatar’s gender (not avatar’s age) reached statistical significance.

**Figure 8:**
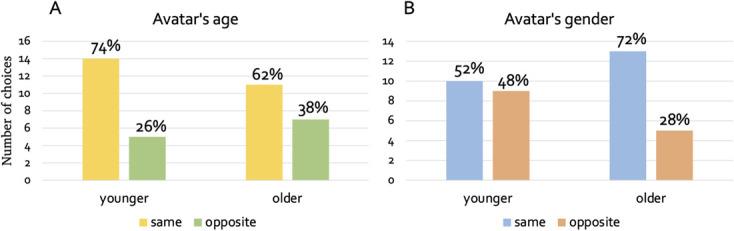
(**A**) Bar plots showing toddler (same age) and adult (opposite age) avatar chosen by younger and older preschoolers. (**A**) Bar plots showing same and opposite-sex avatar chosen by younger and older preschoolers

To explore if there were any differences in the choice of the avatar associated with age, we split the sample between younger and older preschoolers (median=4.31 years). Out of the 19 younger participants, 14 (74%) chose to play with a toddler-avatar and 5 (26%) chose to play with an adult avatar. Out of the 18 older participants, 11 (62%) chose to play with a toddler-avatar and 7 (38%) chose to play with an adult avatar. Out of the 19 younger participants, 10 (52%) chose to play with a toddler-avatar and 9 (48%) chose to play with an adult avatar. Out of the 18 older participants, 13 (72%) chose to play with a toddler-avatar and 5 (28%) chose to play with an adult avatar. A chi-square test of independence showed that there was not a statistically significant association between the participant’s age and the preferred avatar’s age (*X^2^*(1, *N* = 37) = 0.66, *p* = 0.41) (Fig. 8A), and between the participant’s age and the preferred avatar’s gender (*X^2^*(1, *N* = 37) = 1.5, *p* = 0.21) (Fig. 8B). To sum up, there was no statistically significant association between participants’ age and choice of avatar’s gender or age. However, it seems that younger preschoolers preferred to play with the toddler-avatars more than the older ones, and older preschoolers preferred to play with the same-sex avatars more than the younger ones.

**Figure 9:**
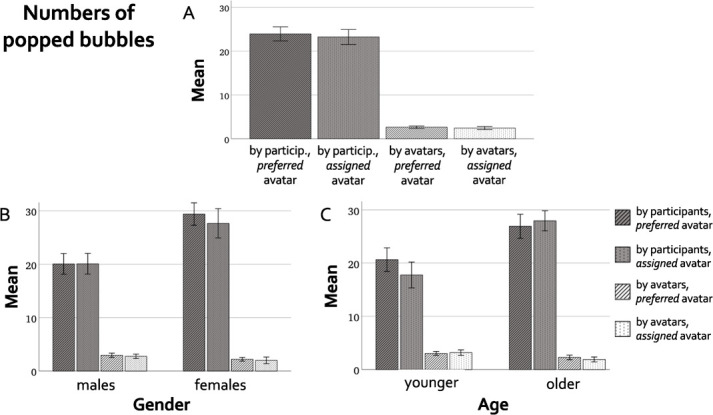
Bar plots showing the number of popped bubbles in the preferred and the assigned avatar condition, both by the participants and by the avatars (paired t-test). (**A**) Whole sample; (**B**) subsamples of female and male participants; (**C**) subsamples of younger and older participants. Error bars are +/– 1 SE

We hypothesized that preschoolers would pop more bubbles in the *preferred avatar* compared to the *assigned avatar* condition. However, a paired t-test showed that there was no difference between the number of bubbles popped when playing with the preferred avatar compared to the assigned avatar (*t*(36) = 0.46, *p* = 0.64) (Fig. 9A). This difference was not statistically significant either in the subsamples of female (*t*(14) = 0.91, *p* = 0.37), male (*t*(21) = 0.01, *p* = 0.99), younger (*t*(18) = 1.1, *p* = 0.28) and older (*t*(17) = 0.59, *p* = 0.55) preschoolers (Fig. 9B and C). Moreover, there was no difference between the number of bubbles popped by the preferred or the assigned avatar, confirming that avatars of different conditions engaged with the game in the same way (*t*(36) = 0.53, *p* = 0.59) (Fig. 9A).

### fNIRS results

**Figure 10:**
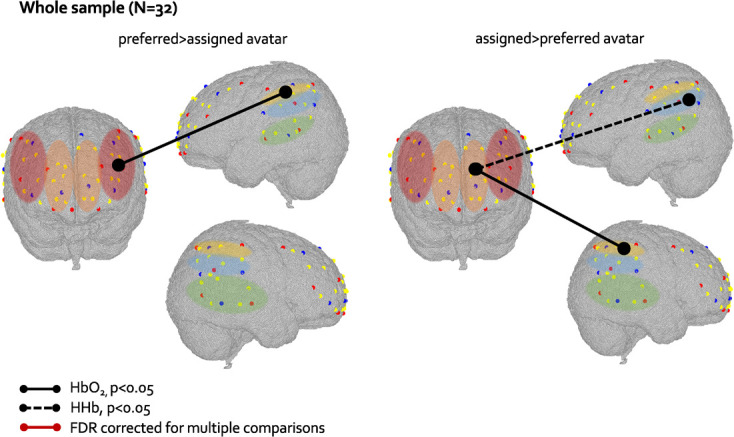
Graphical representation of differences at the paired t-test in FC between preferred and assigned avatar condition in the whole sample. Solid lines represent differences in FC in the HbO_2_ signal and dotted lines represent differences in FC in the HHb signal. Black lines represent FC significant at p<0.05. ROIs are colour-coded as in Fig. 5

32 preschoolers contributed to the fNIRS data. We first explored if connectivity between frontal and temporoparietal regions was engaged in the *preferred avatar* and *assigned avatar* condition by performing a one-sample t-test for each condition. In the HbO_2_ signal, bilateral DLPFC showed significant functional connectivity with bilateral S/MTG, TPJ and IPL, and bilateral MPFC with bilateral S/MTG, TPJ and rIPL both in the *preferred avatar and* in the *assigned avatar* condition. In the HHb signal, all the regions of the front showed significant functional connections with all the temporo-parietal regions in both the *preferred avatar* and *assigned avatar* condition. All these significant functional connections in the HbO_2_ and the HHb signals survived FDR correction for multiple comparisons (see graphical representation of these results in supplementary materials, Fig. S1). A paired t-test showed that there was a stronger lDLPFC-lIPL (*t*(31) = 2.06, *p* = 0.04) connectivity in the *preferred avatar* condition compared to the *assigned avatar* condition in the HbO_2_ signal. Moreover, there was a stronger lMPFC-rIPL (*t*(30) = 2.18, *p* = 0.03) connectivity in the HbO_2_ signal and a stronger lMPFC-lTPJ (*t*(30) = 2.27, *p* = 0.03) connectivity in the HHb signal in the *assigned avatar* condition compared to the *preferred avatar* condition (Fig. 10). However, none of these different FC between *preferred* and *assigned avatar* condition survived FDR correction for multiple comparisons.

**Figure 11:**
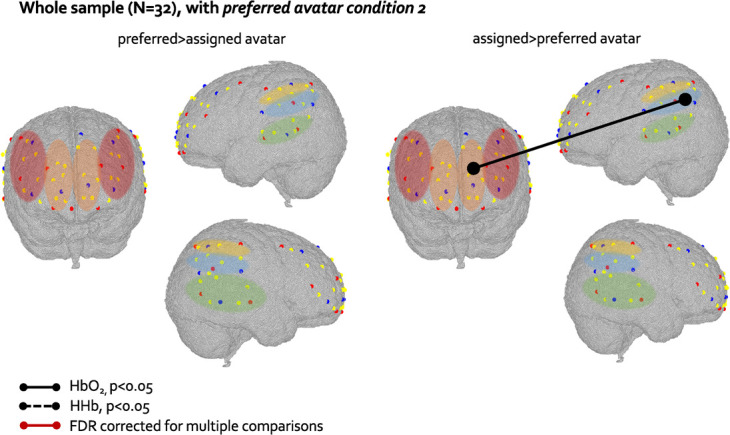
Graphical representation of differences at the paired t-test in FC between preferred and assigned avatar condition in the whole sample, in which we selected the preferred avatar condition 2 for 16 participants. Solid lines represent differences in FC in the HbO_2_ signal significant at p<0.05. ROIs are colour-coded as in Fig. 5

To rule out any condition order effect on FC results, we reperformed the preferred vs. assigned avatar analysis by including the *preferred avatar condition 2* instead of the *preferred avatar condition* in 16 randomly chosen participants out of the 21 who underwent this condition. Therefore, in this analysis, 16 participants contributed with the *preferred avatar condition* firstly presented (followed by the *assigned avatar condition*) and the other 16 with *the preferred avatar condition 2* presented at the end (preceded by the *assigned avatar condition*)*.* Results showed a stronger lMPFC-rTPJ connectivity (*t*(30) = 2.36, *p* = 0.02) in the HbO_2_ signal in the *assigned* compared to *preferred avatar condition* (Fig. 11). This replicated the result found in the HHb signal in the original analysis when considering the preferred condition presented at first for all participants. The lMPFC-rIPL connectivity, which was stronger in the *assigned* compared to *preferred avatar condition* in the HbO_2_ signal in the original preferred vs. assigned avatar analysis (as shown in Fig. 10), showed a trend towards significance in the HbO_2_ signal in this additional analysis (*t*(30) = 1.94, *p* = 0.06). The strength of the lMPFC-lTPJ connectivity, which was stronger in the *assigned* compared to *preferred avatar condition* in the HHb signal in the original preferred vs. assigned avatar analysis (as shown in Fig. 10), was not significantly different in the two conditions in this additional analysis (*t*(30) = 1.38, *p* = 0.1), although showed the same trend as in the original analysis, with greater values in the *assigned* compared to the *preferred avatar condition* in the HHb signal (mean difference 0.1027).

**Figure 12:**
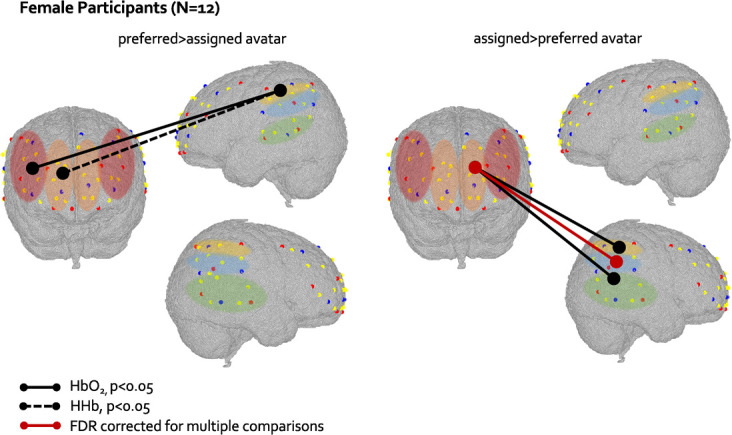
Graphical representation of differences at the paired t-test in FC between preferred and assigned avatar condition in the female sample. Solid lines represent differences in FC in the HbO2 signal and dotted lines represent differences in FC in the HHb signal. Black lines represent FC significant at p<0.05, and red lines represent FC significant and surviving FDR correction. ROIs are colour-coded as in Fig. 5

As only female participants significantly preferred to interact with same-gender and same-age avatar (while male participants did not), we explored if there were any differences in FC between the *preferred* and *assigned avatar* condition in male and female participants separately*.* Males did not show any FC differences between the two conditions. Females showed stronger rDLPFC-lIPL connectivity (*t*(10) = 2.29, *p* = 0.04) in the HbO_2_ signal, and stronger rMPFC-lIPL (*t*(10) = 2.35, *p* = 0.04) in the HHb signal in the *preferred* compared to *assigned avatar* condition. Moreover, females showed stronger lMPFC-rS/MTG (*t*(11) = 2.34, *p* = 0.03), lMPFC-rTPJ connectivity (*t*(11) = 4.51, *p* < 0.001) and lMPFC-rIPL connectivity (*t*(11) = 3.25, *p* = 0.007) in the HbO_2_ signal in the *assigned* compared to *preferred avatar* condition (Fig. 12). Only the lMPFC-rTPJ connectivity stronger in the *assigned* compared to the *preferred avatar condition* survived the FDR correction for multiple comparisons.

Younger and older preschoolers did not show clear difference in the choice of the avatar to play with. We exploratorily investigated any age differences in FC between the two avatar conditions and we presented these results in the supplementary materials (Fig. S2).

## DISCUSSION

Developing new testing platforms to assess preschoolers’ social development is of the paramount importance to advance the field of developmental neuroscience. To date, our understanding of preschoolers development is generally limited, because we lack suitable experimental approaches to test children at an age at which they notably struggle to comply with strict rules of standard lab testing [1]. Moreover, assessing social skills by showing static pictures on a screen does not resemble the dynamic and complexity of children’s social interactions. Therefore, scientists interested in social neurodevelopment have recently started thinking about more naturalistic ways to explore behavioural and neural underpinnings of social interactions, as so far it has been challenging to recreate the complexity of our social lives in traditional lab set-ups [2, 58]. In this work, as a proof-of-principle, we used for the first time a virtual-reality set-up combined with wearable fNIRS to assess partner social preferences in 3-to-5-year-olds. Participants were presented with human-like avatars of different age (adult vs. child) and gender (male vs. female) and were asked to choose one virtual partner to play a bubble-popping game with. While actively engaging and freely playing both with the preferred avatar and a randomly assigned one (i.e. different age or gender to the one preferred one) in a virtual classroom, we used wearable fNIRS to record spontaneous brain fluctuations from the frontal and temporoparietal cortex, known to be engaged in social processing, social interactions, and self-comparison [19, 28, 38, 40, 43, 59].

While virtual-reality has been used with school-age children (for example see [60–62]), for the first time we successfully employed it with preschoolers to investigate their social preference during engaging social exchanges naturalistically. We found that more than 60% of the preschoolers preferred to play with a human-like avatar of their same age and gender. This is consistent with what we hypothesized, as by the 3^rd^ year of life children seem to understand gender differences and segregate into gender-based groups [26]. Moreover, as most children nowadays attend day-care, they interact with other children of their age and not only with their adult carers. Interestingly, we found an association between participants’ gender and choice of the avatar’s age and gender, with only female preschoolers choosing to interact with virtual characters of their same age and gender. While only the association between participants’ gender and choice of avatar’s gender was statistically significant, the pattern observed between participants’ gender and choice of avatar’s age was identical, and likely to reach statistical significance in a bigger sample. As it is possible that participants underwent self-comparison processes when choosing the preferred avatar, this might suggest that females are more advanced in or more sensitive to self-related processes than males. While gender differences in social skills and self-comparison have been documented from childhood through the whole lifespan [37, 63–66], this work provides one of the first evidence of different choice of social partner (and possibly different self-comparison processes) between male and female preschoolers as young 3 years of age. We did not find a significant association between the participants’ age and the choice of the avatars, and the trend of response is unclear, with younger preschoolers prioritizing the avatar’s age and older ones the avatar’s gender. It would have been interesting to split the sample into younger-females, younger-males, older-females, older-males to explore if any difference in social preferences was driven by an interaction of participants’ age and gender. While this investigation was not possible in the current study due to the limited sample size, we invite future research to explore this further.

We successfully integrated wearable fNIRS in the immersive virtual-reality set-up for preschoolers brain imaging assessment. fNIRS data inclusion rate was 86%, which is significantly higher than observed so far in infant fNIRS traditional experiments [67]. Moreover, out of the five participants excluded from the fNIRS analyses, only one was excluded because of excessive motion and noise in the data. The other four were excluded because of experimental errors or poorly fitting cap. Out of the three participants excluded due to a poorly fitting cap, two were males, and their average age was older than the mean age of the participants. This underscores the importance of equipping labs with different cap sizes, which is a limitation that can easily be overcome in future studies. Out of the forty-one toddlers recruited, only four refused to participate in the task and wear the equipment. Interestingly, the majority of these were females, and their average age was younger than the mean age of the participants. This may suggest that young female toddlers might require more time to familiarize with this novel setup or might benefit from a more gradual introduction to the equipment, such as entering the CAVE with the lights off. These findings offer valuable suggestions for future studies utilizing this innovative platform. Moreover, in future studies with this set-up, it may be beneficial collecting information about the child’s prior experience with technologies or their general approach towards strangers, as these factors could potentially influence the time participants need to familiarize with the set-up. Generally, the high inclusion rate of this study highlights the feasibility of using this platform with preschoolers, and the high tolerability of the equipment by this population. More importantly, by collecting high-quality data within this novel platform, we opened up new avenues to explore social development.

Our hypothesis was to find stronger MPFC-TPJ connectivity in the low-frequency range in the *preferred* compared to *assigned avatar* condition. The MPFC-TPJ was indeed the different between the two conditions in the whole sample (in the HHb signal), and survived the FDR correction for multiple comparisons in the female subsample (in the HbO_2_ signal). However, contrary to what hypothesized, this MPFC-TPJ connection was stronger in the *assigned* compared to the *preferred avatar* condition. While both the MPFC and the TPJ have been shown to be activated when interacting with ingroup members, i.e. someone similar to me [38–40, 68], one may think that in young children, who have just developed the ability to perform self-comparison, the mechanism behind the engagement of brain regions related to social categorization might work differently. However, the TPJ region has been found to be more activated for ingroup rather than outgroup members already in 1-year-old infants [19], making this interpretation unlikely. Alternatively, considering that the MPFC-TPJ connectivity have been associated with self-referential processing both in adults and in toddlers [28, 43, 69], it could be that our participants, and in particular females, compared themselves with the virtual character more while they were interacting with the assigned avatar rather than with the preferred one. In fact, at the beginning of the task they chose themselves who they wanted to play with, so any self-categorization and self-comparison process with this chosen character might had happened before the actual play session (and the fNIRS recording) started. Even if the MPFC-TPJ connectivity characterized the interaction with the non-chosen/assigned avatar rather than the preferred one more strongly, this still seems to be a marker of differentiation of a different profile at the neural level between the interactions with the two avatars, which might be happening only in females. The MPFC-TPJ connectivity is significantly stronger in the *assigned* compared to *preferred avatar condition* also when considering the *preferred avatar condition 2* for half of the participants. This suggests that the different FC patterns observed when interacting with the two avatars are likely a genuine effect of the interaction with different social partners and not driven by the condition order effect.

The fact that this different MPFC-TPJ connectivity patterns found when interacting with the two avatars is driven by the female subsample seems to be consistent with the behavioural results, and with several empirical studies documenting gender differences at the neural level, especially in social context [36, 37], and, if replicated, this could be the one of the first evidence of gender differences in neural underpinnings of social interactions in preschoolers. An alternative explanation of our fNIRS findings should acknowledge that the different FC patterns found between the two avatars conditions observed in the female sample only and not in the male one reflects a general difference between males and females brain structure and functions [70–72]. However, we found that both conditions engaged almost all of the social brain regions from which we recorded from in this study in the whole sample, suggesting that the differences found in the female subsample are specific to this task.

In the whole sample (and in the female subsample) we also found greater rDLPFC-lIPL connectivity in the *preferred* compared to the *assigned avatar condition*, which engaged the dorsal portion, and not the medial one as hypothesized, of the frontal cortex. The DLPFC has been more commonly associated with cognitive skills, and especially executive functions [73], which are known to start developing right at the preschool age [74]. The DLPFC-IPL connectivity may possibly belong to the frontoparietal network (FPN), which is known to be involved in sustained attention and goal-oriented cognition [75, 76]. Therefore this FC which might be interpreted as a neural substrate of this conscious choice and differentiation between the preferred and the assigned avatar.

Contrary to what hypothesized, we did not find any difference in the number of bubbles popped by the participants between the *preferred* and *assigned* avatar condition (neither in the whole sample, nor in the subsamples of participants’ different gender or age). This means that on average our preschoolers engaged behaviourally (i.e. popping bubbles) with the two avatars in the same way, regardless of whether they were playing with the chosen or a random avatar, ruling out the possibility that the differences found at the neural level are driven by participants’ movements or engagement with the game.

The absence of behavioural differences in the number of popped bubbles seem to contrast with the fact that all participants exhibited a preference among the avatars, and with the distinct patterns of functional connectivity when interacting with the preferred and assigned avatars found at the group level. In future studies, researchers could model the ingroup-outgroup variable more robustly or ask participants to allocate resources to the avatars, aiming to evoke stronger behavioural differences in the conditions.

One limitation of the present study may be the sample size. Although it is sufficient for pilot and proof-of principle studies [77], future studies might need to recruit more participants to more confidently and reliably assess preschoolers social development, its neural correlates, and any gender- or age-related differences. Moreover, as this was the first study performed with this novel method and equipment, we restricted the choice of the human-like avatar to only two features (age and gender) and we proposed to the children only one activity in one scenario (popping bubbles in a virtual classroom). We acknowledge that the avatars all showed white Caucasian features. However, this matched the ethnicities of all our sample but three participants (two Chinese and one Indian). Further, all participants attended day-care in London, therefore they are exposed to children of different cultural and ethnic backgrounds. In the future, it will be worth exploiting the virtual-reality set-up to its full potential, and assess whether additional features of the virtual characters, such as race, affects preschoolers social preferences, or whether the request of performing different activities, such as problem-solving games, or being in different scenarios, such as in a noisy space, have implications on their social partner choice.

Another possible limitation of this work maybe be the non-counterbalanced order of the two conditions. However, asking half of the participants to play with a randomly assigned avatar first would have then affected their choice of preferred avatar later, as by then they had interacted more with one of the four characters. An alternative solution would have been to make them choose a preferred avatar first, then making them play with a randomly assigned one, and only at the end with the preferred avatar. However, this again would not be comparable to the standard preferred-assigned order, as making the preschoolers choose a character and then playing with another could trigger frustration and confusion in the participants. Discussing these issues when planning a naturalistic study is informative for the field as it highlights some of the new challenges researchers are facing when designing a task that mimic interactions in real life, yet meeting statistical rigour. The overlapping results between the standard preferred vs. assigned analysis and when including the *preferred avatar condition 2* for half of the participants not only gives more confidence in interpreting these results (as they were replicated when using a different *preferred avatar condition* than in the original analysis*)*, but also suggests that the differences in the FC patterns are driven by the modulation of the task and not by the order in which the avatar was presented to the participants.

Despite these limitations, this study provides a proof-of-principle for assessing social development using cutting-edge technologies. For the first time, social neurodevelopment has here been assessed by actively engaging the participants in human-like interactions, rather than passively exposing them to static repeated pictures. There are major limitations in using standard lab set-up for studying social aspects of development and test preschoolers [1], and for the first time we successfully implemented wearable fNIRS with immersive virtual-reality to measure children’s brain functions in a more realistic environment, meeting the need for more dynamic and ecologically valid studies [2]. In the near future, we will validate this novel platform for studying neurodiverse children, with the aim of fine-tuning and personalizing this set-up to better suit their specific needs [80].

One may wonder how much these findings are generalizable to the real world. While performing behavioural assessments and observations in the field is possible and informative to preliminary assess some aspects of child development [23], it is hard to control for noise and confounding variables in these environments. Using an immersive virtual-reality set-up allows to have full control of the experimental variables and to assess the participants in a more dynamic, complex and interactive environment than a traditional screen-based study [10]. In this study, we believe that our participants understood that the virtual scenarios and the human-like avatars are not real, although we took great care to implement important features of realistic social interactions in our avatars (such as eye gaze, engagement with the game, small limb movements while standing, lip-sync while speaking) to resemble real social exchanges. As we continue to use this novel set-up with developmental populations, it would be interesting to explore to which degree toddlers and children understand that this is not real. This is particularly relevant, as it has been shown that children feel more “realness” in an immersive virtual-reality environment than adults [78, 79], findings from studies performed in immersive virtual-reality with developmental population might be more generalizable and similar to what we would have found in the real world than with adults. Taking advantage of this, using novel virtual-reality facilities, equipped with neuroimaging tools, might open up new avenues to rethink about methods to explore social development in preschoolers and children.

## Supplementary Material

Web_Material_kvad012

## Footnotes

1 The array used in this study was designed to cover wider regions than those hypothesized to be engaged by this task, as it will be used for other tasks of the same project.
