# Supplementary material for: Combining wearable fNIRS and immersive virtual reality to study preschoolers’ social development: a proof-of-principle study on preschoolers’ social preference
Source: Oxf Open Neurosci. 2023 Dec 15;2:kvad012. doi: 10.1093/oons/kvad012 (PMC10913823; doi:10.1093/oons/kvad012)
Supplement: Web_Material_kvad012 [file Web_Material_kvad012.zip › Supplementary Materials.pdf]

Supplementary Materials For

Combining wearable fNIRS and immersive virtual reality to study preschoolers' social development: a proof-of-principle study on preschoolers' social preference

This file includes:

FC statistically significant at the one sample t-tests in the preferred and assigned avatar conditions in the whole sample	2
Differences in FC between the preferred avatar and assigned avatar condition in younger and older participants	3

FC statistically significant at the one sample t-tests in the *preferred* and *assigned avatar* conditions in the whole sample

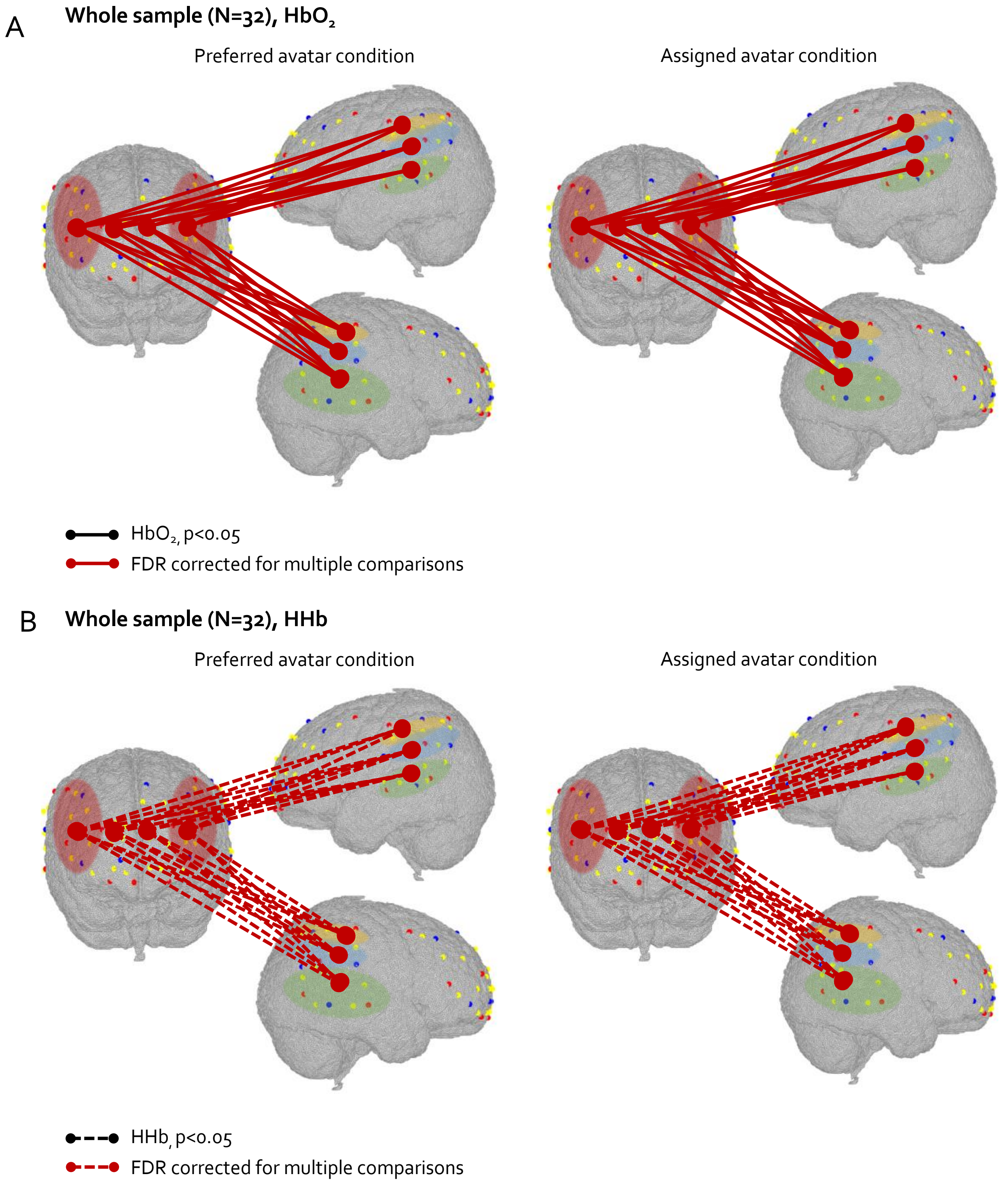

Figure SM1. Graphical representation of the FC statistically significant at the one sample t-tests in the *preferred* and *assigned avatar* conditions in the whole sample, in the HbO_2 signal (A) and in the HHb signal (B), FDR corrected for multiple comparisons. ROIs are colour-coded as in Figure 5.

Differences in FC between the *preferred* avatar and *assigned avatar* condition in younger and older participants

We explored if there were any differences in FC between the *preferred* avatar and *assigned avatar* condition in younger and older participants separately (median=4.31 years). Younger participants showed greater FC between lDLPFC-rIPL ($t(15)$,=2.40, p=0.02), rDLPFC-lIPL ($t(14)$,= 2.41, p=0.02) in the HHb signal in the *preferred* compared to *assigned avatar* condition (Figure SM2A). None of these results survived FDR correction for multiple comparisons.

Older participants showed greater FC between rDLPFC-lIPL ($t(14)$,=2.47, p=0.02), rMPFC-rIPL (t(15),=2.19, p=0.04), lMPFC-lTPJ ($t(14)$,=2.51, p=0.02) in the HbO_2 signal in the *assigned avatar* compared to *preferred* condition (Figure SM2B). All of these results survived FDR correction for multiple comparisons.

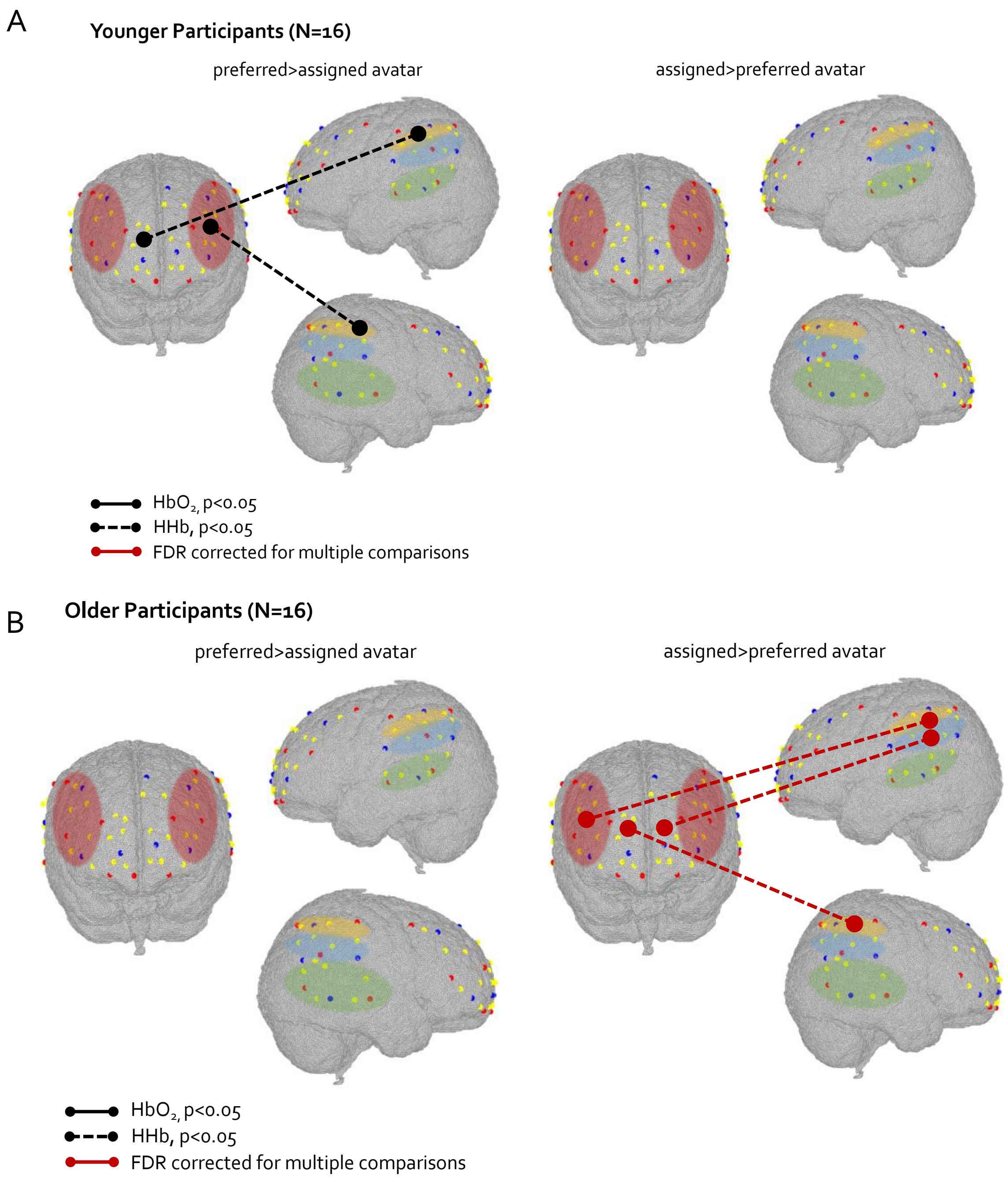

Figure SM2. Graphical representation of differences in FC between *preferred* and *assigned avatar* condition in younger and older participants. Solid lines represent differences in FC in the HbO_2 signal and dotted lines represent differences in FC in the HHb signal. ROIs are colour-coded as in Figure 5.
