# Supplementary material for: Combining wearable fNIRS and immersive virtual reality to study preschoolers’ social development: a proof-of-principle study on preschoolers’ social preference
Source: Oxf Open Neurosci. 2023 Dec 15;2:kvad012. doi: 10.1093/oons/kvad012 (PMC10913823; doi:10.1093/oons/kvad012)
Supplement: Web_Material_kvad012 [file Web_Material_kvad012.zip › Reviewers comments - Supplementary Material - OXFNSC-2023-012.R1.docx]

**REVIEWER COMMENTS**

**OXFNSC-2023-012.R1 -Combining wearable fNIRS and immersive virtual reality to study preschoolers’ social development: a proof-of-principle study on preschoolers’ social preference**

**Original submission - OXFNSC-2023-012**

**Reviewer 1**

Comments to the Author

Dear editorial team and authors,

I was very impressed by this work, not least of all by the specific tailoring of the equipment (glasses, head model for 5-yo etc.) to enable free-play. I found the study methodologically sound and appreciated how the replication with the second preferred avatar condition nicely resolves the question of order effects. I very much enjoyed the discussion, particularly of the functional connectivity findings. I have a handful of contents about the substance the manuscript and further below, many language tips to enhance the readability. All things considered, this work is impressive and should be published once some minor changes are made.

Comments about substance of manuscript:

- INTRO page 4, line 5: It would be really nice to read a sentence or two describing the CAVE visually to the reader, and perhaps referring to the lovely Figure 1A, before learning the pros and cons. A mental image of the amazing set up would help guide the reader.

- INTRO page 5, lines 35-40: There is a switch from females to girls/boys in this sentence. Is this intentional? Perhaps sticking with female/male would increase consistency. Though, it could also be really nice to read “female toddlers” instead of “females” throughout. Food for thought!

- METHODS page 7, line 46: Please spell out LCD.

- METHODS page 8, figure caption: “B) Equipment worn by a participant.” is probably sufficient.

- METHODS page 9, line 28: Do you mean that the avatar in the virtual scenario had the same wand? If so, might be good to state this in similar terms.

- METHODS page 10: I’m not sure what it means where it says “the avatars played with the game and with the participants, indiscriminately…”. Is there maybe another way of phrasing this?

- METHODS page 12, lines 43-51: This came as a surprise, given that the hypotheses only address the MPFC and TPJ. Perhaps a sentence could be added below the hypotheses on page 6 to let the reader know that the surrounding areas will also be included in the analyses. If this is done, then it’s not necessary to explain that additional areas are included in the montage for future work on page 11 (lines 10-14).

- RESULTS page 17, line 6-7: “almost significant” would be better described as a trend without making a qualitative reference to the significance.

- DISCUSSION page 24, line 14: This study doesn’t directly address inclusion rates, although it provides a great example of how to achieve good ones! Therefore, perhaps the authors could rephrase the sentence to say that the inclusion rate “is substantially higher than observed”, and omit the “what”.

- DISCUSSION page 26, lines 1-14: It seems like the behavioural task doesn’t offer great insight into social preference. Perhaps a comment here about how the task could be made more sensitive to social preferences would be useful.

Language and phrasing tips to improve the reader’s experience :)

ABSTRACT

- Line 29: perhaps change to “chose an interaction partner from one of 4 human-like avatars of different gender and age”

- Line 30: “brain fluctuations” (plural)?

- Line 34: “played a bubble-popping game”

- Line 43: “feasibility” instead of “feasibly”. But also, would we expect functional connectivity not to be modulate-able in this environment. Perhaps the phrasing could be more like “We showed that preschoolers social preference can be assessed at the level of behaviour and functional connectivity using this novel set-up/platform” (platform if you want to be consistent with the wording above).

LAY ABSTRACT

- Line 18: Consider changing 37 to “Thirty-seven”.

- Line 19: “of different ages and genders” (plural would be lovely here). I would also recommend starting the next sentence with “The toddlers/children” instead of “they”, and consider using “a bubble-popping game” throughout, if you like.

- Line 23: “with an avatar of a similar age and gender to them” might be more specific

- Line 27: “when interacting with the chosen vs. assigned avatars” might make this clearer

INTRODUCTION page 3

- Line 16: “ with the added risk…” would flow a tiny bit better

- Line 21: either commas or brackets around “typically very young…”, not both :)

- Line 27: “sitting still and paying attention”

- Line 39: perhaps “responses from participants” instead of “reactions in”

- Line 53: perhaps “neural activity” rather than “correlates”, because correlates usually comes with “of x”

- Line 57: perhaps “tethered to stationary machines” rather than “big”

INTRODUCTION page 4

- Line 30: “no empirical studies that investigate partner preference” instead of “investigated”

- Line 41: “the mechanisms supporting/underpinning/underscoring/enabling” instead of “behind” and “have yet to be fully understood” or “remain poorly understood” perhaps

- Line 44: “preferences in social partners, suggesting…”

- Line 45: consider using “look longer/more frequently at/ are more attentive to..” instead of just “look more”

- Line 51: “individuals/people they perceive as more similar to them”

- Line 60: “to inform our understanding of children’s..”

INTRODUCTION page 5

- Line 23: no comma before “and the capability…”

- Line 35: “vs.”

INTRODUCTION page 6:

- Line 9: maybe write out “second” instead of “2nd”

- Line 18: write out 41 in letters for readability

- Line 34: “of the same age and gender as themselves” to clarify that the hypothesis isn’t “they all pick one avatar”

- Line 35: “assigned avatar condition, indexing social preferences;” or something similar to clarify the measure. Though, it could also be good to put that “indexing..” in front of both hypotheses 2/3, because it applies to both.

- Line 48: “extremely novel, as we used …” perhaps without “for the first time”

METHODS page 7:

- Line 7: Write out 37 in letters

- Line 11: “fNIRS cap or complete the task” if you prefer

- Line 16: write out 32 in letters

- Line 20: no space in “full-term”

- Line 50: dash in “optical motion-tracking cameras”

- Line 51: “Passive markers for head tracking were attached..” might add flow

METHODS page 8:

- Line 15: Consider using dash in “toddler girl-avatar” and whether girl or female is desired

METHODS page 9:

- Line 42: one more comma “(a boy, and a girl)”

- Line 49: I would go with “Thereafter” but it’s not the end of the world

- Line 51: “randomly assigned from those…”

- Line 56: I would take out “issues”: “… might create order effects related to the preferred avatar always being first.”

METHODS page 10:

- Line 14: Perhaps “we believe” could be replaced with “we collected data for 3 minutes…”

METHODS page 11:

- Line 9: I would consider flipping the parts of the sentence beginning with: “Although we had clear…” as “This array was designed to cover broader regions than those needed for this proof-of-principle (MPFC-TPJ) as the data will be reused in other studies.”

METHODS page 12:

- Line 12: commas before and after “channels, based on physiological indicators of quality, were…”

- Line 27: no comma needed

- Line 28: “surviving/remaining” and comma needed “inspection, and channels…”

- Line 32: no comma needed

METHODS page 13:

- Line 40: comma before “, as well as …”

RESULTS page 18:

- Line 26: “We hypothesized that preschoolers would pop more bubbles…”

- Figure captions: for al figures showing brains, “Figure 5.” could be capitalized on last line of caption.

DISCUSSION page 23:

- Line 25: move naturalistically to the end of the sentence: “to investigate…social exchanges naturalistically.”

- Line 33: no comma needed between “age, and not”

DISCUSSION page 24:

- Line 19: plural “experimental errors or poorly fitting caps”

- Line 26: comma “this novel platform, we have opened up…”

DISCUSSION page 25:

- Line 6-7: “strongly” at the end of the clause, “connectivity characterized the interaction with … than the preferred one more strongly, …”

- Line 51: “putative” isn’t quite the right word “plausibly” or “possibly” would be better

DISCUSSION page 26:

- Line 21: “…, its neural correlates and any gender- or age-related differences.” would be sufficient 

- Line 53: it would be good to add “one/avatar” to the end of the sentence “preferred one/avatar”.

DISCUSSION page 27:

- Line 19: dash in “cutting-edge”

- Line 40-41: replace “yet” with “… variables and to assess the participant…”. Also “interacting” should probably be “interactive”.

- Line 49: limb not plural “small limb movements”

- Line 51: Why not phrase this “As we continue to use this novel set-up with developmental populations….”

- Line 54: Perhaps start the sentence with “This is particularly relevant as it has been shown….”

**Reviewer 2**

Comments to the Author

Authors presented a virtual reality set-up combined with a functional near-infrared spectroscopy (fNIRS) to record spontaneous brain fluctuation from frontal and temporoparietal regions while preschoolers played a “popping bubbles” game with a preferred and an assigned avatar. Authors report that most preschoolers preferred to play with an avatar similar to them (i.e., age, sex) and they exhibited different connectivity patterns among temporoparietal regions while interacting with the two different avatars. It is also intriguing that the avatar identity did not moderate performance of ‘popping bubbles.’ This study provides the first feasibility data for using such technologies and naturalistic experiments to study social development, potentially enabling new lines of research in social development. This is an interesting and novel study. The manuscript was written very clearly and the results were straightforward. I offer two major suggestions to address (which could be discussed), and minor points to clarify the statistics and text:

Major

1) I understand that participants were excluded because 4 refused to wear the fNIRS hat and do the task and 3 were excluded because the hat didn’t fit properly. This does suggest that participants may not have been acclimated properly to the headgear. In fact, I could not find details on how long the children were allowed to adjust to the hat. The actual details are vague: (line 23, page 34) “… the participant was made familiar with the CAVE set-up…” and (line 37, page 34) “Initially, the participants had some time to explore the virtual classroom…” Could authors provide some more detailed information on how long, on average, the children were permitted to familiarize themselves with the set-up? Were there gender differences? Authors should note the criteria used, if any, to determine the participants were suitably familiarized.

2) It is possible that preschoolers differ in terms of what parents have conveyed about adult strangers- some could trust them more than others. Have authors considered trust of strange adults / potential gender differences in that variable as moderator(s) to consider in these studies in the future? Additionally, preschoolers could differ in their experience with technology- a parent survey could address this and maybe could be described in limitations/future direction in the Discussion. Similarly, authors should seek to potentially outline more explicit exclusion criteria to rule out variables that may confound the outcomes of interest. For example, there could be neuropsychological battery/assessments to provide more information about the sample in various relevant cognitive (attention) and socioemotional domains. Will authors include neurodivergent participants in such social development studies in the future? Authors should provide more details on these issues.

Minor

Indicate the statistical tests used for Figures 7-12 captions.

Figure 9 text is very small and the axes labels and legends are tiny- please correct.

Line 43, page 4: “… makes it very challenging to unpick complex causal pathways between the behaviour of interacting partners.” Unpack or tease apart?

**Decision letter - OXFNSC-2023-012**

13-Nov-2023

Dear Dr. Bulgarelli,

Manuscript ID OXFNSC-2023-012 entitled "Combining wearable fNIRS and immersive virtual reality to study preschoolers’ social development: a proof-of-principle study on preschoolers’ social preference" which you submitted to the Oxford Open Neuroscience, has been reviewed. The comments of the reviewer(s) are included at the bottom of this letter.

The reviewer(s) have recommended publication, but also suggest some revisions to your manuscript prior to acceptance. Therefore, I invite you to respond to the reviewer(s)' comments and revise your manuscript within the next 30 days of todays date.

Please note that this journal operates with transparent peer review. This means that if your submission is accepted for publication, the full peer review history of your article will be published online alongside your article. This includes reviewer comments, editor decision letters, and your author responses.

To revise your manuscript, log into https://mc.manuscriptcentral.com/oxfnsc and enter your Author Centre, where you will find your manuscript title listed under "Manuscripts with Decisions." Under "Actions," click on "Create a Revision." Your manuscript number has been appended to denote a revision.

You may also click the below link to start the revision process (or continue the process if you have already started your revision) for your manuscript. If you use the below link you will not be required to login to ScholarOne Manuscripts.

*** PLEASE NOTE: This is a two-step process. After clicking on the link, you will be directed to a webpage to confirm. ***

https://mc.manuscriptcentral.com/oxfnsc?URL_MASK=c4f0cb4c85b1438e978ee6ca09b9c9ac

You will be unable to make your revisions on the originally submitted version of the manuscript. Instead, revise your manuscript using a word processing program and save it on your computer. Please also highlight the changes to your manuscript within the document by using the track changes mode in MS Word or by using bold or colored text.

Once the revised manuscript is prepared, you can upload it and submit it through your Author Centre.

When submitting your revised manuscript, you will be able to respond to the comments made by the reviewer(s) in the space provided. You can use this space to document any changes you make to the original manuscript. In order to expedite the processing of the revised manuscript, please be as specific as possible in your response to the reviewer(s).

IMPORTANT: Your original files are available to you when you upload your revised manuscript. Please delete any redundant files before completing the submission.

Because we are trying to facilitate timely publication of manuscripts submitted to the Oxford Open Neuroscience, your revised manuscript should be uploaded within the next 30 days from todays date. If it is not possible for you to submit your revision in a reasonable amount of time, we may have to consider your paper as a new submission.

Once again, thank you for submitting your manuscript to the Oxford Open Neuroscience and I look forward to receiving your revision.

Sincerely,

Dr. Alicia Izquierdo

Senior Editor, Oxford Open Neuroscience

aizquie@psych.ucla.edu

Reviewer: 1

Comments to the Author

Dear editorial team and authors,

I was very impressed by this work, not least of all by the specific tailoring of the equipment (glasses, head model for 5-yo etc.) to enable free-play. I found the study methodologically sound and appreciated how the replication with the second preferred avatar condition nicely resolves the question of order effects. I very much enjoyed the discussion, particularly of the functional connectivity findings. I have a handful of contents about the substance the manuscript and further below, many language tips to enhance the readability. All things considered, this work is impressive and should be published once some minor changes are made.

Comments about substance of manuscript:

- INTRO page 4, line 5: It would be really nice to read a sentence or two describing the CAVE visually to the reader, and perhaps referring to the lovely Figure 1A, before learning the pros and cons. A mental image of the amazing set up would help guide the reader.

- INTRO page 5, lines 35-40: There is a switch from females to girls/boys in this sentence. Is this intentional? Perhaps sticking with female/male would increase consistency. Though, it could also be really nice to read “female toddlers” instead of “females” throughout. Food for thought!

- METHODS page 7, line 46: Please spell out LCD.

- METHODS page 8, figure caption: “B) Equipment worn by a participant.” is probably sufficient.

- METHODS page 9, line 28: Do you mean that the avatar in the virtual scenario had the same wand? If so, might be good to state this in similar terms.

- METHODS page 10: I’m not sure what it means where it says “the avatars played with the game and with the participants, indiscriminately…”. Is there maybe another way of phrasing this?

- METHODS page 12, lines 43-51: This came as a surprise, given that the hypotheses only address the MPFC and TPJ. Perhaps a sentence could be added below the hypotheses on page 6 to let the reader know that the surrounding areas will also be included in the analyses. If this is done, then it’s not necessary to explain that additional areas are included in the montage for future work on page 11 (lines 10-14).

- RESULTS page 17, line 6-7: “almost significant” would be better described as a trend without making a qualitative reference to the significance.

- DISCUSSION page 24, line 14: This study doesn’t directly address inclusion rates, although it provides a great example of how to achieve good ones! Therefore, perhaps the authors could rephrase the sentence to say that the inclusion rate “is substantially higher than observed”, and omit the “what”.

- DISCUSSION page 26, lines 1-14: It seems like the behavioural task doesn’t offer great insight into social preference. Perhaps a comment here about how the task could be made more sensitive to social preferences would be useful.

Language and phrasing tips to improve the reader’s experience :)

ABSTRACT

- Line 29: perhaps change to “chose an interaction partner from one of 4 human-like avatars of different gender and age”

- Line 30: “brain fluctuations” (plural)?

- Line 34: “played a bubble-popping game”

- Line 43: “feasibility” instead of “feasibly”. But also, would we expect functional connectivity not to be modulate-able in this environment. Perhaps the phrasing could be more like “We showed that preschoolers social preference can be assessed at the level of behaviour and functional connectivity using this novel set-up/platform” (platform if you want to be consistent with the wording above).

LAY ABSTRACT

- Line 18: Consider changing 37 to “Thirty-seven”.

- Line 19: “of different ages and genders” (plural would be lovely here). I would also recommend starting the next sentence with “The toddlers/children” instead of “they”, and consider using “a bubble-popping game” throughout, if you like.

- Line 23: “with an avatar of a similar age and gender to them” might be more specific

- Line 27: “when interacting with the chosen vs. assigned avatars” might make this clearer

INTRODUCTION page 3

- Line 16: “ with the added risk…” would flow a tiny bit better

- Line 21: either commas or brackets around “typically very young…”, not both :)

- Line 27: “sitting still and paying attention”

- Line 39: perhaps “responses from participants” instead of “reactions in”

- Line 53: perhaps “neural activity” rather than “correlates”, because correlates usually comes with “of x”

- Line 57: perhaps “tethered to stationary machines” rather than “big”

INTRODUCTION page 4

- Line 30: “no empirical studies that investigate partner preference” instead of “investigated”

- Line 41: “the mechanisms supporting/underpinning/underscoring/enabling” instead of “behind” and “have yet to be fully understood” or “remain poorly understood” perhaps

- Line 44: “preferences in social partners, suggesting…”

- Line 45: consider using “look longer/more frequently at/ are more attentive to..” instead of just “look more”

- Line 51: “individuals/people they perceive as more similar to them”

- Line 60: “to inform our understanding of children’s..”

INTRODUCTION page 5

- Line 23: no comma before “and the capability…”

- Line 35: “vs.”

INTRODUCTION page 6:

- Line 9: maybe write out “second” instead of “2nd”

- Line 18: write out 41 in letters for readability

- Line 34: “of the same age and gender as themselves” to clarify that the hypothesis isn’t “they all pick one avatar”

- Line 35: “assigned avatar condition, indexing social preferences;” or something similar to clarify the measure. Though, it could also be good to put that “indexing..” in front of both hypotheses 2/3, because it applies to both.

- Line 48: “extremely novel, as we used …” perhaps without “for the first time”

METHODS page 7:

- Line 7: Write out 37 in letters

- Line 11: “fNIRS cap or complete the task” if you prefer

- Line 16: write out 32 in letters

- Line 20: no space in “full-term”

- Line 50: dash in “optical motion-tracking cameras”

- Line 51: “Passive markers for head tracking were attached..” might add flow

METHODS page 8:

- Line 15: Consider using dash in “toddler girl-avatar” and whether girl or female is desired

METHODS page 9:

- Line 42: one more comma “(a boy, and a girl)”

- Line 49: I would go with “Thereafter” but it’s not the end of the world

- Line 51: “randomly assigned from those…”

- Line 56: I would take out “issues”: “… might create order effects related to the preferred avatar always being first.”

METHODS page 10:

- Line 14: Perhaps “we believe” could be replaced with “we collected data for 3 minutes…”

METHODS page 11:

- Line 9: I would consider flipping the parts of the sentence beginning with: “Although we had clear…” as “This array was designed to cover broader regions than those needed for this proof-of-principle (MPFC-TPJ) as the data will be reused in other studies.”

METHODS page 12:

- Line 12: commas before and after “channels, based on physiological indicators of quality, were…”

- Line 27: no comma needed

- Line 28: “surviving/remaining” and comma needed “inspection, and channels…”

- Line 32: no comma needed

METHODS page 13:

- Line 40: comma before “, as well as …”

RESULTS page 18:

- Line 26: “We hypothesized that preschoolers would pop more bubbles…”

- Figure captions: for al figures showing brains, “Figure 5.” could be capitalized on last line of caption.

DISCUSSION page 23:

- Line 25: move naturalistically to the end of the sentence: “to investigate…social exchanges naturalistically.”

- Line 33: no comma needed between “age, and not”

DISCUSSION page 24:

- Line 19: plural “experimental errors or poorly fitting caps”

- Line 26: comma “this novel platform, we have opened up…”

DISCUSSION page 25:

- Line 6-7: “strongly” at the end of the clause, “connectivity characterized the interaction with … than the preferred one more strongly, …”

- Line 51: “putative” isn’t quite the right word “plausibly” or “possibly” would be better

DISCUSSION page 26:

- Line 21: “…, its neural correlates and any gender- or age-related differences.” would be sufficient 

- Line 53: it would be good to add “one/avatar” to the end of the sentence “preferred one/avatar”.

DISCUSSION page 27:

- Line 19: dash in “cutting-edge”

- Line 40-41: replace “yet” with “… variables and to assess the participant…”. Also “interacting” should probably be “interactive”.

- Line 49: limb not plural “small limb movements”

- Line 51: Why not phrase this “As we continue to use this novel set-up with developmental populations….”

- Line 54: Perhaps start the sentence with “This is particularly relevant as it has been shown….”

Reviewer: 2

Comments to the Author

Authors present a virtual reality set-up combined with a functional near-infrared spectroscopy (fNIRS) to record spontaneous brain fluctuation from frontal and temporoparietal regions while preschoolers played a “popping bubbles” game with a preferred and an assigned avatar. Authors report that most preschoolers preferred to play with an avatar similar to them (i.e., age, sex) and they exhibited different connectivity patterns among temporoparietal regions while interacting with the two different avatars. It is also intriguing that the avatar identity did not moderate performance of ‘popping bubbles.’ This study provides the first feasibility data for using such technologies and naturalistic experiments to study social development, potentially enabling new lines of research in social development. This is an interesting and novel study. The manuscript was written very clearly and the results were straightforward. I offer two major suggestions to address (which could be discussed), and minor points to clarify the statistics and text:

Major

1) I understand that participants were excluded because 4 refused to wear the fNIRS hat and do the task and 3 were excluded because the hat didn’t fit properly. This does suggest that participants may not have been acclimated properly to the headgear. In fact, I could not find details on exactly how long the children were allowed to adjust to the hat. The actual details are vague: (line 23, page 34) “… the participant was made familiar with the CAVE set-up…” and (line 37, page 34) “Initially, the participants had some time to explore the virtual classroom…” Could authors provide some more detailed information on how long, on average, the children were permitted to familiarize themselves with the set-up? Were there gender differences? Authors should note the criteria used, if any, to determine the participants were suitably familiarized.

2) It is possible that preschoolers differ in terms of what parents have conveyed about adult strangers- some could trust them more than others. Have authors considered trust of strange adults / potential gender differences in that variable as moderator(s) to consider in these studies in the future? Additionally, preschoolers could differ in their experience with technology- a parent survey could address this and maybe could be described in limitations/future direction in the Discussion. Similarly, authors should seek to potentially outline more explicit exclusion criteria to rule out variables that may confound the outcomes of interest. For example, there could be neuropsychological battery/assessments to provide more information about the sample in various relevant cognitive (attention) and socioemotional domains. Will authors include neurodivergent participants in such social development studies in the future? Authors should provide more details on these issues.

Minor

Indicate the statistical tests used for Figures 7-12 captions.

Figure 9 text is very small and the axes labels and legends are tiny- please correct.

Line 43, page 4: “… makes it very challenging to unpick complex causal pathways between the behaviour of interacting partners.” Unpack or tease apart?

**Author response**

Dear Editor,

We are very grateful to the reviewers for their helpful suggestions to improve our work. We have addressed all the issues raised and we have modified the manuscript accordingly (changes in the manuscript are in red). Below are our responses to the reviewers’ comments.

Reviewer #1

Dear editorial team and authors,

I was very impressed by this work, not least of all by the specific tailoring of the equipment (glasses, head model for 5-yo etc.) to enable free-play. I found the study methodologically sound and appreciated how the replication with the second preferred avatar condition nicely resolves the question of order effects. I very much enjoyed the discussion, particularly of the functional connectivity findings. I have a handful of contents about the substance the manuscript and further below, many language tips to enhance the readability. All things considered, this work is impressive and should be published once some minor changes are made.

We thank the reviewer for the interest and the positive feedback on our work.

Comments about substance of manuscript:

1. INTRO page 4, line 5: It would be really nice to read a sentence or two describing the CAVE visually to the reader, and perhaps referring to the lovely Figure 1A, before learning the pros and cons. A mental image of the amazing set up would help guide the reader.

We agree with the reviewer that this section would benefit from a short description of the CAVE, especially considering the novel of this platform. We have therefore added this information as follow: The CAVE is a unique virtual-reality room able to simulate real-world surroundings. Virtual scenarios are projected on the three walls surrounding the participant and the floor.

2. INTRO page 5, lines 35-40: There is a switch from females to girls/boys in this sentence. Is this intentional? Perhaps sticking with female/male would increase consistency. Though, it could also be really nice to read “female toddlers” instead of “females” throughout. Food for thought!

We thank the reviewer for flagging this, we changed the term “females” with “girl” in the sentence.

3. METHODS page 7, line 46: Please spell out LCD.

We have added the full term for LCD as follow: (i.e. liquid crystal display).

4. METHODS page 8, figure caption: “B) Equipment worn by a participant.” is probably sufficient.

We have modified the caption of the Figure 1B as suggested.

5. METHODS page 9, line 28: Do you mean that the avatar in the virtual scenario had the same wand? If so, might be good to state this in similar terms.

We agree with the reviewer that this sentence might be misleading. The golden wand in the scenario represents the golden wand moved by the participant. We rephrased it as follow: the same golden wand was present in the virtual scenario to represent the participant’s wand, and it moved in sync with the participant's real wand.

6. METHODS page 10: I’m not sure what it means where it says “the avatars played with the game and with the participants, indiscriminately…”. Is there maybe another way of phrasing this?

We agree with the reviewer that this sentence is not very clear. We have replaced it with: To make the task more realistic and interesting for the participants, the avatars played with the game and with the participants, regardless the condition (preferred or assigned avatar).

7. METHODS page 12, lines 43-51: This came as a surprise, given that the hypotheses only address the MPFC and TPJ. Perhaps a sentence could be added below the hypotheses on page 6 to let the reader know that the surrounding areas will also be included in the analyses. If this is done, then it’s not necessary to explain that additional areas are included in the montage for future work on page 11 (lines 10-14).

As the reviewer suggested, we added a footnote on page 6 after the hypothesis as follow: The array used in this study was designed to cover wider regions than those hypothesized to be engaged by this task, as it will be used for other tasks of the same project. We therefore removed the sentence to explain the recording from the additional areas on page 11.

8. RESULTS page 17, line 6-7: “almost significant” would be better described as a trend without making a qualitative reference to the significance.

As the reviewer suggested, we replaced “almost significant” with “a trend towards a significant association”.

9. DISCUSSION page 24, line 14: This study doesn’t directly address inclusion rates, although it provides a great example of how to achieve good ones! Therefore, perhaps the authors could rephrase the sentence to say that the inclusion rate “is substantially higher than observed”, and omit the “what”.

We removed “what” from the sentence as the reviewer suggested.

10. DISCUSSION page 26, lines 1-14: It seems like the behavioural task doesn’t offer great insight into social preference. Perhaps a comment here about how the task could be made more sensitive to social preferences would be useful.

We added this sentence as follow: The absence of behavioural differences in the number of popped bubbles seem to contrast with the fact that all participants exhibited a preference among the avatars, and with the distinct patterns of functional connectivity when interacting with the preferred and assigned avatars found at the group level. In future studies, researchers could model the ingroup-outgroup variable more robustly or ask participants to allocate resources to the avatars, aiming to evoke stronger behavioural differences in the conditions.

Language and phrasing tips to improve the reader’s experience :)

ABSTRACT

11. Line 29: perhaps change to “chose an interaction partner from one of 4 human-like avatars of different gender and age”

12. Line 30: “brain fluctuations” (plural)?

13. Line 34: “played a bubble-popping game”

14. Line 43: “feasibility” instead of “feasibly”. But also, would we expect functional connectivity not to be modulate-able in this environment. Perhaps the phrasing could be more like “We showed that preschoolers social preference can be assessed at the level of behaviour and functional connectivity using this novel set-up/platform” (platform if you want to be consistent with the wording above).

LAY ABSTRACT

15. Line 18: Consider changing 37 to “Thirty-seven”.

16. Line 19: “of different ages and genders” (plural would be lovely here). I would also recommend starting the next sentence with “The toddlers/children” instead of “they”, and consider using “a bubble-popping game” throughout, if you like.

17. Line 23: “with an avatar of a similar age and gender to them” might be more specific

18. Line 27: “when interacting with the chosen vs. assigned avatars” might make this clearer

INTRODUCTION page 3

19. Line 16: “ with the added risk…” would flow a tiny bit better

20. Line 21: either commas or brackets around “typically very young…”, not both :)

21. Line 27: “sitting still and paying attention”

22. Line 39: perhaps “responses from participants” instead of “reactions in”

23. Line 53: perhaps “neural activity” rather than “correlates”, because correlates usually

comes with “of x”

24. Line 57: perhaps “tethered to stationary machines” rather than “big”

INTRODUCTION page 4

25. Line 30: “no empirical studies that investigate partner preference” instead of “investigated”

26. Line 41: “the mechanisms supporting/underpinning/underscoring/enabling” instead

of “behind” and “have yet to be fully understood” or “remain poorly understood” perhaps

27. Line 44: “preferences in social partners, suggesting…”

28. Line 45: consider using “look longer/more frequently at/ are more attentive to..” instead of just “look more”

29. Line 51: “individuals/people they perceive as more similar to them”

30. Line 60: “to inform our understanding of children’s..”

INTRODUCTION page 5

31. Line 23: no comma before “and the capability…”

32. Line 35: “vs.”

INTRODUCTION page 6:

33. Line 9: maybe write out “second” instead of “2nd”

34. Line 18: write out 41 in letters for readability

35. Line 34: “of the same age and gender as themselves” to clarify that the hypothesis isn’t “they all pick one avatar”

36. Line 35: “assigned avatar condition, indexing social preferences;” or something similar to clarify the measure. Though, it could also be good to put that “indexing..” in front of both hypotheses 2/3, because it applies to both.

37. Line 48: “extremely novel, as we used …” perhaps without “for the first time”

METHODS page 7:

38. Line 7: Write out 37 in letters

39. Line 11: “fNIRS cap or complete the task” if you prefer

40. Line 16: write out 32 in letters

41. Line 20: no space in “full-term”

42. Line 50: dash in “optical motion-tracking cameras”

43. Line 51: “Passive markers for head tracking were attached..” might add flow

METHODS page 8:

44. Line 15: Consider using dash in “toddler girl-avatar” and whether girl or female is desired

METHODS page 9:

45. Line 42: one more comma “(a boy, and a girl)”

46.Line 49: I would go with “Thereafter” but it’s not the end of the world

47. Line 51: “randomly assigned from those…”

48. Line 56: I would take out “issues”: “… might create order effects related to the preferred avatar always being first.”

METHODS page 10:

49. Line 14: Perhaps “we believe” could be replaced with “we collected data for 3 minutes…”

METHODS page 11:

50. Line 9: I would consider flipping the parts of the sentence beginning with: “Although we had clear…” as “This array was designed to cover broader regions than those needed for this proof-of-principle (MPFC-TPJ) as the data will be reused in other studies.”

METHODS page 12:

51. Line 12: commas before and after “channels, based on physiological indicators of quality, were…”

52. Line 27: no comma needed

53. Line 28: “surviving/remaining” and comma needed “inspection, and channels…”

54. Line 32: no comma needed

METHODS page 13:

55. Line 40: comma before “, as well as …”

RESULTS page 18:

56. Line 26: “We hypothesized that preschoolers would pop more bubbles…”

57. Figure captions: for all figures showing brains, “Figure 5.” could be capitalized on last line of caption.

DISCUSSION page 23:

58. Line 25: move naturalistically to the end of the sentence: “to investigate…social exchanges naturalistically.”

59. Line 33: no comma needed between “age, and not”

DISCUSSION page 24:

60. Line 19: plural “experimental errors or poorly fitting caps”

61. Line 26: comma “this novel platform, we have opened up…”

DISCUSSION page 25:

62. Line 6-7: “strongly” at the end of the clause, “connectivity characterized the interaction with … than the preferred one more strongly, …”

63. Line 51: “putative” isn’t quite the right word “plausibly” or “possibly” would be better

DISCUSSION page 26:

64. Line 21: “…, its neural correlates and any gender- or age-related differences.” would be sufficient

65. Line 53: it would be good to add “one/avatar” to the end of the sentence “preferred one/avatar”.

DISCUSSION page 27:

66. Line 19: dash in “cutting-edge”

67. Line 40-41: replace “yet” with “… variables and to assess the participant…”. Also “interacting” should probably be “interactive”.

68. Line 49: limb not plural “small limb movements”

69. Line 51: Why not phrase this “As we continue to use this novel set-up with developmental populations….”

70. Line 54: Perhaps start the sentence with “This is particularly relevant as it has been shown….”

We are incredibly grateful to the reviewer for thoroughly revising our manuscript and providing suggestions to improve the readiness of the work. All the reviewer's suggestions, from point 11 to 70, have been addressed/modified as suggested in the manuscript.

Reviewer #2

Authors present a virtual reality set-up combined with a functional near-infrared spectroscopy (fNIRS) to record spontaneous brain fluctuation from frontal and temporoparietal regions while preschoolers played a “popping bubbles” game with a preferred and an assigned avatar. Authors report that most preschoolers preferred to play with an avatar similar to them (i.e., age, sex) and they exhibited different connectivity patterns among temporoparietal regions while interacting with the two different avatars. It is also intriguing that the avatar identity did not moderate performance of ‘popping bubbles.’ This study provides the first feasibility data for using such technologies and naturalistic experiments to study social development, potentially enabling new lines of research in social development. This is an interesting and novel study. The manuscript was written very clearly and the results were straightforward. I offer two major suggestions to address (which could be discussed), and minor points to clarify the statistics and text:

We thank the reviewer for the positive comment on our work.

Major

1. I understand that participants were excluded because 4 refused to wear the fNIRS hat and do the task and 3 were excluded because the hat didn’t fit properly. This does suggest that participants may not have been acclimated properly to the headgear. In fact, I could not find details on exactly how long the children were allowed to adjust to the hat. The actual details are vague: (line 23, page 34) “… the participant was made familiar with the CAVE set-up…” and (line 37, page 34) “Initially, the participants had some time to explore the virtual classroom…” Could authors provide some more detailed information on how long, on average, the children were permitted to familiarize themselves with the set-up? Were there gender differences? Authors should note the criteria used, if any, to determine the participants were suitably familiarized.

The reviewer is correct in noting that we did not provide specific details about the duration during which participants familiarized themselves with the fNIRS cap and the CAVE. This was intentional and based on the dynamics of the testing sessions. We prioritized responsiveness to toddlers' and parents' indications, allowing the parent to tell us when their child was ready to wear the equipment and begin the testing session. In our experience of testing infants and toddlers with neurotechnologies, some individuals may require more time than others to acclimate to the equipment we use. We reflected this in the manuscript as follow (added text is in italics):

- At the beginning of the experiment, the participant was made familiar with the CAVE set-up and the equipment. We allowed the parent and the toddler to explore the fNIRS cap and the CAVE room for as long as they wished. The parent informed the experimenter when their child was ready to wear the equipment and begin the testing session. While there was variability in the time participants took to acclimate to the equipment — with some individuals requiring more time than others — in general, participants were ready to commence the study after approximately 5 minutes of exploring the equipment.

- Initially, the participant had some time to explore the virtual classroom and pop some bubbles with the wand. The time spent on this phase varied among participants, averaging approximately 3 to 5 minutes. This duration depended on the participants' preference to explore the virtual classroom and the time needed for them to successfully pop the bubbles with the magic wand.

In addition, we would like to specify our attrition rate is notably low, considerably less than what observed in other fNIRS studies involving developmental populations, as noted in the discussion. It is interesting, however, to explore more carefully excluded participants’ characteristics of age and gender. The table below summarises this information:

Reason for exclusion N Gender Mean in years (mean ±std)

Refusing to wear the equipment 4 3 F, 1 M 4.07±0.34

Hat did not fit properly 3 1 F, 2 M 4.77±0.93

While these numbers are low for a study involving a developmental population, it is interesting to note that the majority of participants who refused to wear the equipment were females and younger compared to the mean age of the participants (i.e., 4.42 years). Conversely, participants excluded because the hat did not fit properly were primarily males and older than the mean age of the participants. We thank the reviewer for commenting on this, as we think that including this information in the manuscript will be remarkably useful for readers who wants to do studies with similar equipment in the future.

Therefore, we added excluded participants’ gender and age in the participants section as follow (added text in italics):

- 4 additional participants were recruited but refused to wear the fNIRS hat and do the task (1 male, age mean 4.07±0.34 years)

- the fNIRS cap did not fit on their head (3 participants, 2 males, age mean 4.77±0.93 years).

We have also commented on this in the discussion, as follow (added text is in italics): out of the five participants excluded from the fNIRS analyses, only one was excluded because of excessive motion and noise in the data. The other four were excluded because of experimental errors or poorly fitting cap. Out of the three participants excluded due to a poorly fitting cap, two were males, and their average age was older than the mean age of the participants. This underscores the importance of equipping labs with different cap sizes, which is a limitation that can easily be overcome in future studies. Out of the forty-one toddlers recruited, only four refused to participate in the task and wear the equipment. Interestingly, the majority of these were females, and their average age was younger than the mean age of the participants. This may suggest that young female toddlers might require more time to familiarise with this novel setup or might benefit from a more gradual introduction to the equipment, such as entering the CAVE with the lights off. These findings offer valuable suggestions for future studies utilizing this innovative platform.

2. It is possible that preschoolers differ in terms of what parents have conveyed about adult strangers- some could trust them more than others. Have authors considered trust of strange adults / potential gender differences in that variable as moderator(s) to consider in these studies in the future? Additionally, preschoolers could differ in their experience with technology- a parent survey could address this and maybe could be described in limitations/future direction in the Discussion. Similarly, authors should seek to potentially outline more explicit exclusion criteria to rule out variables that may confound the outcomes of interest. For example, there could be neuropsychological battery/assessments to provide more information about the sample in various relevant cognitive (attention) and socioemotional domains. Will authors include neurodivergent participants in such social development studies in the future? Authors should provide more details on these issues.

To outline more clearly exclusion criteria for our study, we have added this information in the participants section, as follow: Participants were excluded from recruitment if they had a known significant neurodevelopmental condition or a medical condition that was likely to impact brain development or impede the child's ability to participate in this study.

The variables highlighted by the reviewer are interesting factors that we have not explored for the current study, but that we are indeed considering the collection of information on in our future works. We added information on this as the end of the discussion, as follow:

- page 25, when discussing excluded participants: In future studies with this set-up, it may be beneficial collecting information about the child’s prior experience with technologies or their general approach towards strangers, as these factors could potentially influence the time participants need to familiarise with the set-up.

- Page 29, when discussing future studies: In the near future, we will validate this novel platform for studying neurodiverse children, with the aim of fine-tuning and personalising this set-up to better suit their specific needs.

Minor

3. Indicate the statistical tests used for Figures 7-12 captions.

We added ‘statistically significant difference at the chi-square’ in the caption of Figure 7. We added paired t-test in the caption of Figure 9. We added ‘differences at the paired t-test in FC...’ in the caption of Figure 10, 11 and 12.

4. Figure 9 text is very small and the axes labels and legends are tiny- please correct.

We replaced the text in Figure 9, enlarging the fonts for both axes and legends, as suggested.

5. Line 43, page 4: “… makes it very challenging to unpick complex causal pathways between the behaviour of interacting partners.” Unpack or tease apart?

We thank the reviewer for spotting this typo, we replaced this with ‘tease apart’.

**Revised Submission - OXFNSC-2023-012.R1**

**Decision letter - OXFNSC-2023-008.R1**

22-Nov-2023

Dear Dr. Bulgarelli,

It is a pleasure to accept your revised manuscript entitled "Combining wearable fNIRS and immersive virtual reality to study preschoolers’ social development: a proof-of-principle study on preschoolers’ social preference" in its current form for publication in the Oxford Open Neuroscience. The comments of the reviewer(s) who reviewed your manuscript are included at the foot of this letter.

Please note that this journal operates with transparent peer review. This means that the full peer review history of your article will publish online alongside your article. This includes reviewer comments, editor decision letters, and your author responses.

Next steps

You will receive an email from no-reply@scipris.com within roughly one week. This is your invitation to sign up for an account with SciPris, Oxford University Press’ author portal hosted by Aptara. You will need to create an account if you do not already hold one. Please register or log into your account and follow the online instructions which will guide you through signing your licence and paying the APC. The email and the portal have clearly signposted support options if you need any help during this process.

Please note that SciPris is a completely different system from ScholarOne, so your credentials to submit your manuscript here will not work there. Once you’ve created a SciPris account, you will be able to use it whenever you publish with Oxford Open Neuroscience or any OUP journal. Please note that OUP will only ever request payment for applicable fees be made via SciPris or to an OUP bank account. If you ever have concerns about the legitimacy of a request, please do not hesitate to contact a customer services agent via the SciPris portal or directly via oupsupport@scipris.com.

Thank you for your fine contribution. On behalf of the Editors of the Oxford Open Neuroscience, we look forward to your continued contributions to the Journal.

Sincerely,

Dr. Alicia Izquierdo

Senior Editor, Oxford Open Neuroscience

aizquie@psych.ucla.edu

Reviewer: 1

Comments to the Author

The manuscript is of very high quality, as is the contribution to the field. I recommend this manuscript be accepted.

Reviewer: 2

Comments to the Author

The authors have well-addressed my comments and suggestions. This is an interesting study with high potential impact in the study of social preference development using wearable technology and virtual reality.

Associate Editor

Comments to the Author:

Reviewers were satisfied with revisions.
